# Bacterial divisome protein FtsA forms curved antiparallel double filaments upon binding FtsN

**DOI:** 10.1038/s41564-022-01206-9

**Published:** 2022-09-19

**Authors:** Tim Nierhaus, Stephen H McLaughlin, Frank Bürmann, Danguole Kureisaite-Ciziene, Sarah L Maslen, J Mark Skehel, Conny WH Yu, Stefan MV Freund, Louise FH Funke, Jason W Chin, Jan Löwe

**Affiliations:** 1 MRC Laboratory of Molecular Biology, Cambridge, UK

## Abstract

During bacterial cell division, filaments of tubulin-like FtsZ form the Z-ring, which is the cytoplasmic scaffold for divisome assembly. In *Escherichia coli*, actin homologue FtsA anchors the Z-ring to the membrane and recruits divisome components, including bitopic FtsN. FtsN regulates the periplasmic peptidoglycan synthase FtsWI. To characterize how FtsA regulates FtsN, we applied electron microscopy to show that *E. coli* FtsA forms antiparallel double filaments on lipid monolayers when bound to the cytoplasmic tail of FtsN. Using X-ray crystallography, we demonstrate that *Vibrio maritimus* FtsA crystallizes as an equivalent double filament. We identified an FtsA-FtsN interaction site in FtsA’s IA-IC interdomain cleft by X-ray crystallography and confirmed that FtsA forms double filaments *in vivo* by site-specific cysteine cross-linking. FtsA-FtsN double filaments reconstituted in or on liposomes prefer negative Gaussian curvature, like those of MreB, the elongasome’s actin. We propose that curved, antiparallel FtsA double filaments together with treadmilling FtsZ filaments organise septal peptidoglycan synthesis in the division plane.

## Introduction

In non-spherical, walled bacteria, cell shape is determined by a load-bearing structure counteracting turgor pressure named the peptidoglycan sacculus^1^. Insertion of newly synthesised glycan strands into the sacculus is mediated by the divisome in cell division, and by the elongasome (or rod complex) in cell elongation^2^. In *Escherichia coli*, both of these multiprotein complexes span the entire cell envelope, and contain the cytoplasmic, membrane-binding and filament-forming actin homologues FtsA (divisome) and MreB (elongasome)^3,4^.

In *E. coli* and many other bacteria, FtsA is the main membrane anchor for the cell division ring, the Z-ring, which initiates and organises division^5^. ZipA can function as an alternative membrane anchor for the Z-ring but seems to have a minor role in cell division in unperturbed cells^5^. The Z-ring is mainly formed by filaments of the tubulin homologue FtsZ^6–8^. FtsZ treadmilling dynamics, driven by FtsZ’s GTPase activity, were shown to be essential for the initial condensation of FtsZ filaments into the Z-ring, but seem to become dispensable after partial constriction of the septum^9–11^.

Apart from localising FtsZ to the membrane, FtsA is also involved in the recruitment of essential divisome proteins such as FtsK, FtsN and potentially FtsQ^12,13^. Together with the bitopic membrane protein FtsN, FtsA forms an interaction that is crucial for the recruitment of divisome components^14^. Most of FtsN assembles last into the divisome and activates or regulates the bipartite septal PG synthase FtsWI via FtsQLB^15–19^. Small amounts of FtsN are recruited early to the divisome in a FtsA-dependent manner^20^.

FtsA has a uniquely positioned IC domain among actin homologues^21^. FtsA’s IC domain is important for the interaction with the cytoplasmic tail of FtsN, which comprises ~32 amino acids in *E. coli*^22–26^. Two FtsN suppressor mutations in FtsA located in its IC domain^16,27^ further support the idea that FtsN binds the IC domain of FtsA. In FtsN, a conserved stretch of basic amino acids in its N-terminal, cytoplasmic tail is required for interaction with FtsA *in vitro*^22,24^. In a *zipA* null background, in which the FtsA-FtsN interaction becomes essential, mutation of the basic stretch in FtsN was however permissible, whereas a D5N mutation was not^23^. Taken together with previous yeast two-hybrid assays^28^, it was suggested that interaction with FtsN depolymerises FtsA and, thereby, allows recruitment of downstream divisome components via binding sites that were (partially) occluded in the FtsA polymer^14,23^. FRET microscopy found the hypermorphic FtsA* mutant to be less polymeric than wildtype FtsA on supported lipid bilayers^29^. Addition of cytoplasmic FtsN peptide in those experiments however induced FtsA* polymerisation to levels comparable to wildtype FtsA, challenging the previous model that FtsN depolymerises FtsA.

Despite the uniqueness of FtsA’s IC domain amongst the actin-like proteins^21^, FtsA was shown to form single protofilaments that recapitulate some structural features of *bona fide* actin protofilaments^30^. Double filaments were shown to be the smallest functional unit of all other known actin homologues^31^ and, indeed, *Thermotoga maritima* FtsA was also shown to form double filaments^30^. More recently, several mutations in *E. coli* FtsA, originally described as ZipA suppressor mutations, were shown to facilitate double filament formation of FtsA on supported lipid monolayers^32^.

Similar to FtsA, *E. coli* MreB, the actin-like protein of the elongasome, binds membranes directly through an amphipathic helix and forms curved antiparallel double filaments^4,33,34^. This enables a curvature-sensing mechanism that allows MreB filaments to align with the axis of highest principal curvature, the short axis of the cell in the case of rod-shaped cells, such as *Bacillus subtilis*^34,35^. Hence, the elongasome uses MreB double filaments as “rudders” to direct movement of its bipartite PG synthase RodA-PBP2 (similar to FtsWI in the divisome) and, thereby, directs insertion of new PG strands around the cell circumference^36^. It is thought that the radially inserted PG hoops enforce rod shape by mechanically limiting cell width expansion.

Here, we investigated how FtsA functions in the divisome in bacteria using structural and genetic approaches. We characterise the interaction between *E. coli* FtsA and FtsN and its effect on the architecture of FtsA double filaments. We propose a curvature-based mechanism by which FtsA filaments contribute to oriented peptidoglycan synthesis.

### *Vibrio maritimus* FtsA crystallises as an antiparallel double filament

**Extended Data Figure 1:**
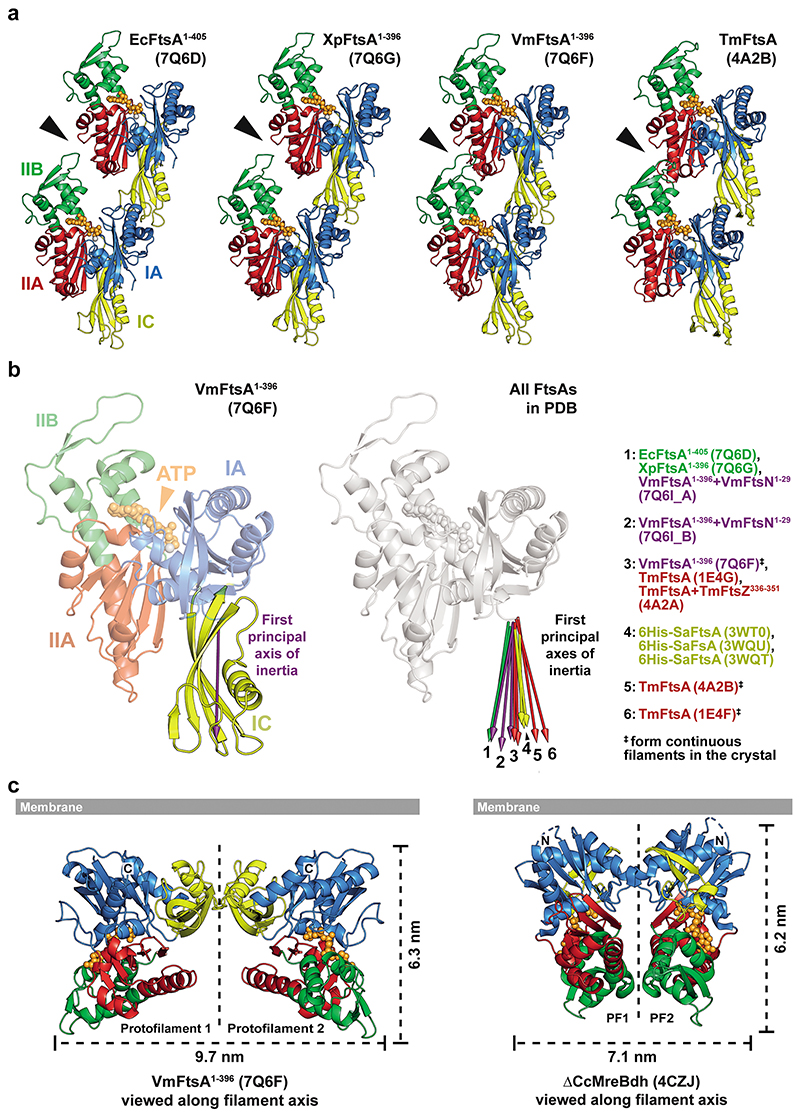
The position of the IC domain within the FtsA monomer varies. **a**, Comparison of longitudinal filament contacts in FtsA crystal structures from *E. coli* (PDB 7Q6D), *X. poinarii* (PDB 7Q6G), *V. maritimus* (PDB 7Q6F) and *T. maritima* (PDB 4A2B). *E. coli* and *X. poinarii* FtsA form “loose” protofilaments with detached IIA and IIB domains (arrowhead). IIA and IIB domains are in close contact in the VmFtsA and TmFtsA structures, which form continuous filaments in the crystals. **b**, The IC domain of FtsA is flexible. Left: an arrow along the first principal axis of inertia of the IC domain (purple) can be used to indicate IC domain orientation. Right: FtsA structures in the PDB aligned on their IA, IIA and IIB domains, with arrows indicating the position of the IC domains, showing that the IC domain orientation is variable within the FtsA monomer. There is no correlation between IC domain position and species (different colours) or formation of continuous filaments in the crystals (^‡^). Principal axes of inertia were calculated using main chain atoms (N, CA, C) of IC domains. **c**, Comparison between the FtsA and MreB double filaments. Because the lateral interface is formed by the IC domain in the FtsA double filament, it is wider than the MreB double filament. The membrane proximal side of both double filaments is flat.

**Figure 1:**
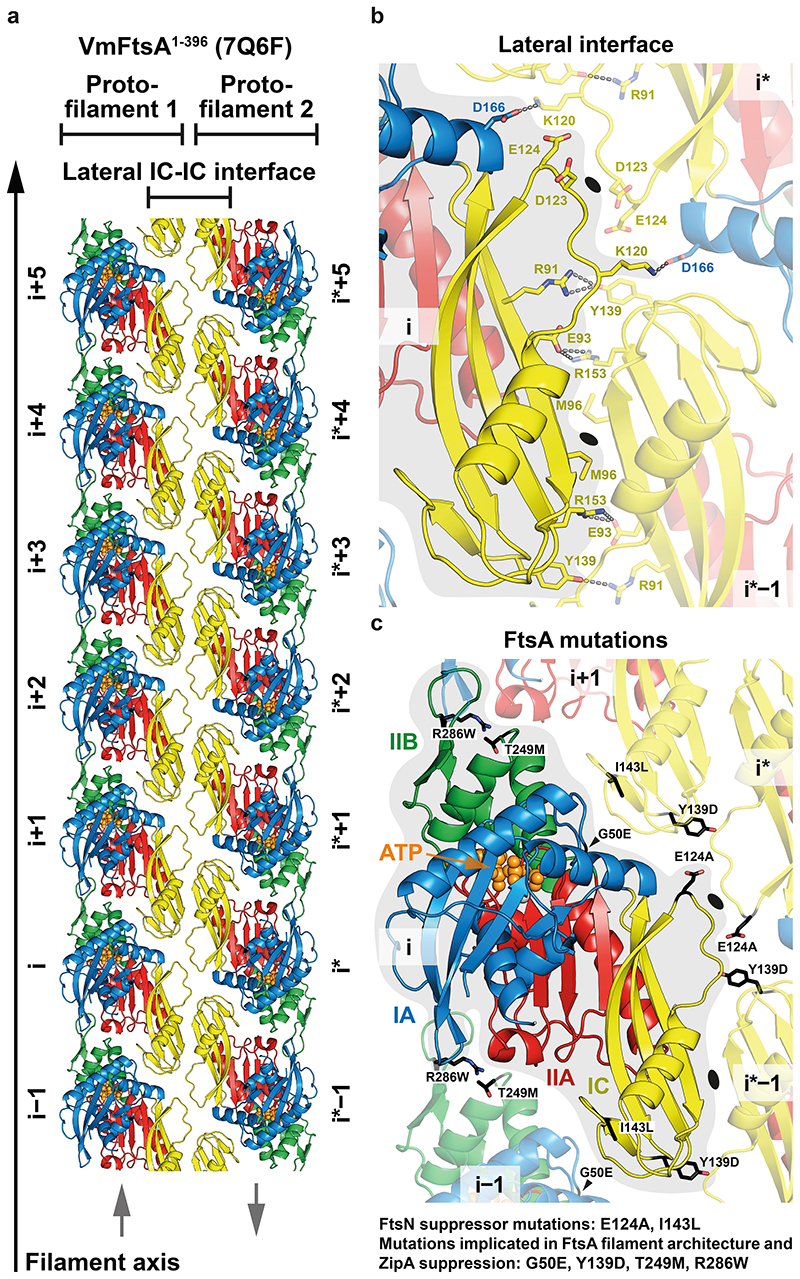
VmFtsA crystallises as an antiparallel double filament via IC domains. **a**, Top view of the VmFtsA^1-396^ antiparallel double filament in cartoon representation from the membrane-proximal side (PDB 7Q6F). The lateral interface is almost exclusively formed by the IC domain (yellow). Grey arrows indicate the relative orientations of FtsA monomers in each protofilament. **b**, Side chain interactions in the lateral filament interface. Each FtsA_i_ contacts two neighbouring FtsAs, FtsA_i*-1_ and FtsA_i*_, in the opposing protofilament. Both interfaces have local C2 symmetry (black ellipses). **c**, Reported FtsN suppressor mutations in *E. coli* FtsA, E124A^27^ and I143L^16^, and ZipA suppressor mutations that recently have been implicated in FtsA (double) filament formation^32^ are mapped on the VmFtsA double filament structure. All mutations are part of filament interfaces. For further information about the suppressor mutants see Supplementary Table T2.

We solved the crystal structures of C-terminally truncated FtsAs from *E. coli* (EcFtsA, Protein Data Bank (PDB) identifier: 7Q6D) and the closely related Gram-negative bacteria *Xenorhabdus poinarii* (PDB 7Q6G, 90 % identity of XpFtsA to EcFtsA) and *Vibrio maritimus* (PDB 7Q6F, 70 % identity of VmFtsA to EcFtsA) (Supplementary Table T1). The EcFtsA^1-405^ and XpFtsA^1-396^ structures showed single protofilaments with a “loose” longitudinal contact between the IIA and IIB subdomains (Extended Data Figure 1a). In contrast, VmFtsA^1-396^ crystallised as straight, antiparallel double filaments, with lateral contacts formed by the IC domain, which among actins is unique to FtsA^21^ (Figure 1a). The longitudinal interface contact between the IIA-IIB domains was “tight” in both protofilaments, as was the case previously for TmFtsA bound to ATP-γ-S (PDB 4A2B)^30^ (Extended Data Figure 1a). We noticed that the position of the IC domain is variable within FtsA monomers across FtsA structures (Extended Data Figure 1b). Positioning of the IC domain does neither correlate with species nor the presence of continuous protofilaments within the crystal, as analysed for PDB 1E4F, 4A2B and 7Q6F.

The lateral interface in the VmFtsA double filament is almost exclusively formed by contacts between IC domains (Figure 1b). Each FtsA monomer contacts two subunits in the opposing protofilament, forming the FtsA_i_-FtsA_i*_ and FtsA_i_-FtsA_i*−1_ lateral interfaces. Both interfaces have local twofold (C2) symmetry as indicated in Figure 1b. The VmFtsA double filament is wider than the MreB double filament (PDB 4CZJ)^33^, which is, like FtsA filaments, comprised of two antiparallel protofilaments (Extended Data Figure 1c). The membrane-proximal side of FtsA and MreB double filaments is flat. ZipA suppressor mutations in *E. coli* FtsA (including FtsA^R286W^, also known as FtsA*), which were described to also affect filament architecture^32^ (Supplementary Table T2), correspond to residues in subunit interfaces in the VmFtsA double filament structure (Figure 1c). Interestingly, the FtsN suppressor mutations E124A^27^ and I143L^16^ described for *E. coli* FtsA also map to subunit interfaces in the VmFtsA double filament structure (Figure 1c).

### *E. coli* FtsA forms antiparallel double filaments with EcFtsN^1-32^

**Extended Data Figure 2:**
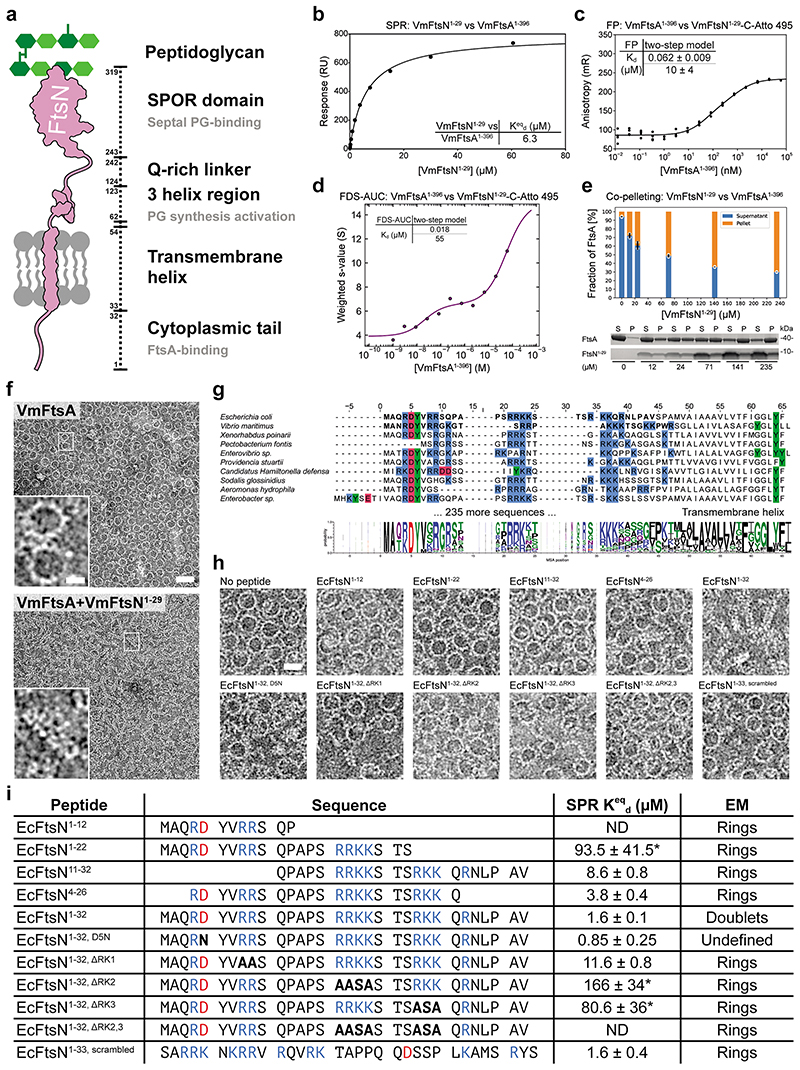
VmFtsA forms antiparallel double filaments upon binding VmFtsN^1-29^. **a**, Schematic overview of EcFtsN. **b**, SPR equilibrium response titration of VmFtsN^1-29^ binding to immobilised VmFtsA^1-396^. Binding affinity is about three fold lower than for the EcFtsA^1-405^-EcFtsN^1-32^ interaction (Figure 2b). **c**, VmFtsA^1-396^ titration into VmFtsN^1-29^-C-Atto 495. Data were fitted with a two step model, with transitions being indicative of FtsN binding and polymerisation (panel d). A representative quadruplicate is shown. K_d_s are given as mean ± SEM from five independent experiments. **d**, Weight-averaged sedimentation coefficients of a VmFtsA^1-396^ titration into VmFtsN^1-29^-C-Atto 495 by FDS-AUC shows that VmFtsN^1-29^ is part of higher order FtsA polymers. Data were fitted to a two step model, recapitulating the FP data in panel c. **e**, Co pelleting assay of VmFtsN^1-29^ titrated into VmFtsA^1-396^ indicates that VmFtsN^1-29^ induces FtsA polymerisation. A representative SDS-PAGE gel is shown. Given are mean ± sd (black lines) of technical duplicates (white dots). P: pellet, S: supernatant. **f**, Negative stain electron micrographs of VmFtsA with and without VmFtsN^1-29^ on supported lipid monolayers. VmFtsA forms “mini-rings” in the absence of FtsN and double filaments at ten fold molar excess of VmFtsN^1-29^. Two independent grids were examined per condition. Scale bar, 50 nm, 20 nm (inset). **g**, Multiple sequence alignment of 245 FtsN sequences comprising cytoplasmic and transmembrane domains. EcFtsN^1-32^ and VmFtsN^1-29^ sequences are highlighted in bold. **h**, Mapping of the FtsA-interacting region in EcFtsN^1-32^ using the lipid monolayer assay. EcFtsN^1-32^ mutants were in ten fold molar excess of FtsA. In contrast to EcFtsN^1-32^, EcFtsN^1-32, D5N^ and a scrambled version of the EcFtsN^1-32^ peptide^24^ did not induce FtsA double filaments. EcFtsN^4-26^ and EcFtsN^1-32, ΔRK1^ led to formation of fewer double filaments. At least two independent grids were examined per condition. Scale bar, 20 nm. **i**, Summary of EcFtsN^1-32^ peptides. Mutations are highlighted in bold. Equilibrium dissociation constants (K^eq^_d_) are given as mean ± SEM (n ≥ 2 for each construct). For weak binders the maximum response was fixed during fitting, hence these are only approximate values as indicated by asterisks. ND: not determinable. The predominant higher order polymer observed in the monolayer assay is given in the “EM” column. Note that EcFtsN^4-26^ and EcFtsN^1-32, ΔRK1^ still lead to formation of a few FtsA double filaments.

**Figure 2:**
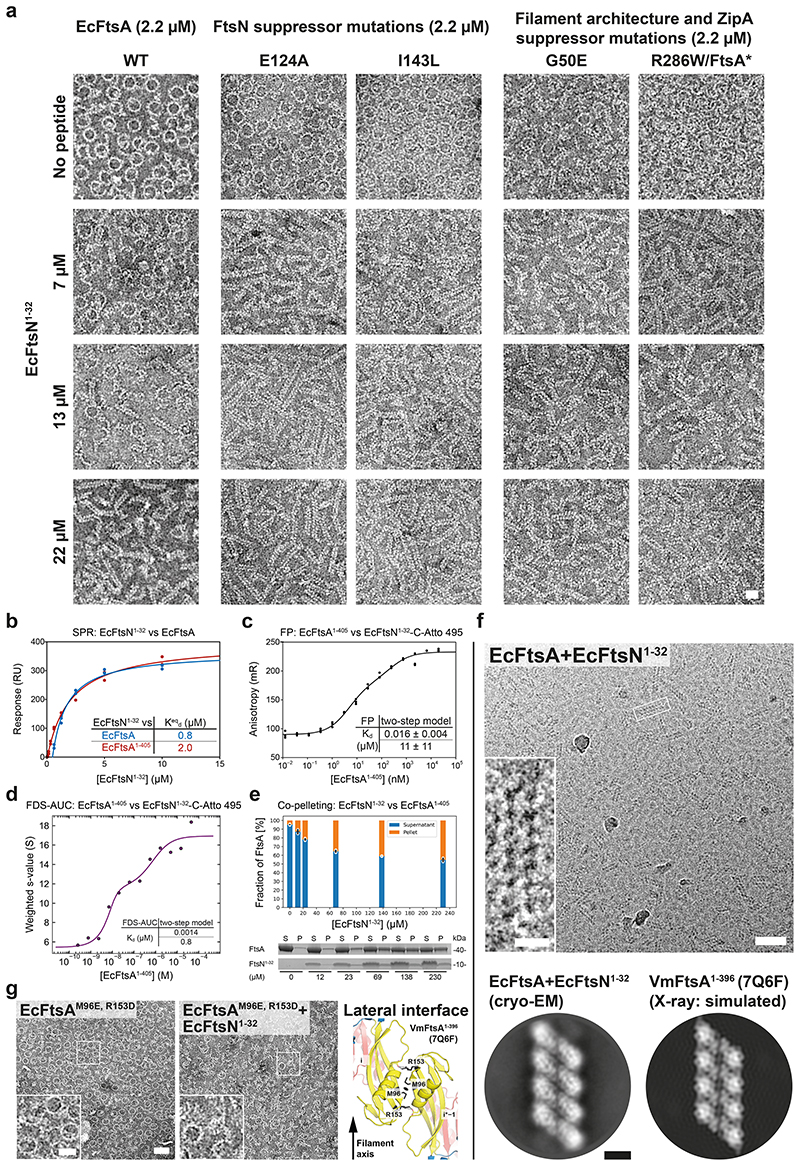
EcFtsA forms antiparallel double filaments upon binding EcFtsN^1-32^. **a**, Negative stain electron micrographs of EcFtsA mutants on supported lipid monolayers with increasing concentrations of EcFtsN^1-32^. EcFtsA forms “mini-rings” in the absence of FtsN peptide, as described previously^37^. With increasing EcFtsN^1-32^ concentrations, EcFtsA forms fewer “mini-rings” and more double filaments. Introduction of the G50E, E124A, I143L or R286W mutations into EcFtsA facilitates filament formation at lower concentrations of EcFtsN^1-32^. See also Figure 1c and Supplementary Table T2. At least two independent grids were examined per condition. Scale bar, 20 nm. **b**, SPR equilibrium response titration of EcFtsN^1-32^ binding to immobilised EcFtsA (blue) or EcFtsA^1-405^ (red). EcFtsN^1-32^ has micromolar affinity for both FtsA proteins. **c**, EcFtsA^1-405^ titration into EcFtsN^1-32^-C-Atto 495. Data were fitted with a two-step model, with transitions being indicative of FtsN binding and polymerisation (panel d). A representative triplicate is shown. K_d_s are given as mean ± SEM from five independent experiments. **d**, Weight-averaged sedimentation coefficients of a EcFtsA^1-405^ titration into fluorescently labelled EcFtsN^1-32^-C-Atto 495 by FDS-AUC shows that EcFtsN^1-32^ is part of higher order FtsA polymers. Data were fitted to a two-step model, recapitulating the FP data in panel c. **e**, Co-pelleting assay of EcFtsN^1-32^ titrated into EcFtsA^1-405^ indicates that EcFtsN^1-32^ induces FtsA polymerisation. A representative SDS-PAGE gel is shown. Given are mean ± sd (black lines) of technical duplicates (white dots). P: pellet, S: supernatant. **f**, EcFtsA and EcFtsN^1-32^ form double filaments on lipid monolayers as determined by cryo-EM. 2D classification confirmed the antiparallel arrangement of the observed double filaments. A computed 2D projection from the VmFtsA double filament crystal structure (PDB 7Q6F) is shown for comparison. Two independent grids were examined. Data was collected on one grid. Scale bars, 50 nm, 10 nm (inset), 5 nm (2D class averages). **g**, The lateral interface mutant EcFtsA^M96E^, ^R153D^ is deficient in FtsN^1-32^-dependent double filament formation as determined by negative stain electron microscopy on a supported lipid monolayer. Two independent grids were examined per condition. Scale bar, 50 nm, 20 nm (inset).

As described previously^37^, we found that *E. coli* FtsA forms “mini-rings” on supported lipid monolayers (Figure 2a, “WT + no peptide”). Given that filament architecture mutations and FtsN suppressor mutations in *E. coli* FtsA map to lateral interfaces in the VmFtsA double filament (Figure 1c), we hypothesised that FtsN could affect double filament formation of *E. coli* FtsA. The short, cytoplasmic tail of FtsN (comprising ~32 amino acids in *E. coli*) (Extended Data Figure 2a) interacted with FtsA as shown previously^20,22,23^. Using surface plasmon resonance (SPR), we showed that EcFtsN^1-32^ binds EcFtsA and EcFtsA^1-405^, a C-terminal truncation of EcFtsA lacking the amphipathic membrane binding helix, with dissociation constants (K_d_) of 0.8 μM and 2.0 μM, respectively (Figure 2b). The interaction between *V. maritimus* FtsA^1-396^ and FtsN^1-29^ was about three-fold weaker than the EcFtsA^1-405^-EcFtsN^1-32^ interaction (Extended Data Figure 2b). Since cross-linking of FtsA to the flow cell surface in SPR might affect FtsA polymerisation, we also probed the FtsA-FtsN interaction in solution using fluorescence polarisation (FP), for which C-terminally truncated FtsA was titrated into Atto 495-labelled FtsN peptide. FP data were fitted to a two-step model with K_d_s of about 0.016 μM and 11 μM for the EcFtsA^1-405^-FtsN^1-32-C-Atto 495^ interaction (Figure 2c). Again, the VmFtsA^1-396^-FtsN^1-29-C-Atto 495^ interaction was about four-fold weaker for the first binding event (Extended Data Figure 2c). Next, we subjected the system to analytical ultracentrifugation using a fluorescence detection system (FDS-AUC) to characterise the two binding events of the FtsA-FtsN interaction. FDS-AUC showed that both binding events are accompanied by formation of higher order FtsA-FtsN assemblies, indicating that the cytoplasmic FtsN peptide not only binds to FtsA but could also facilitate FtsA polymerisation (Figure 2d; Extended Data Figure 2d). Using a co-pelleting assay, for which cytoplasmic FtsN peptide was titrated into truncated FtsA, we showed that FtsN peptide indeed induces FtsA polymerisation (Figure 2e; Extended Data Figure 2e).

Given that the cytoplasmic peptide of FtsN induces FtsA polymerisation, we next investigated the architecture of those polymers using negative stain electron microscopy (EM). Both EcFtsA-FtsN^1-32^ and VmFtsA-FtsN^1-29^ formed short double filaments on supported lipid monolayers (Figure 2a, “WT”; Extended Data Figure 2f). Electron cryo-microscopy (cryo-EM) imaging and subsequent 2D averaging of EcFtsA-FtsN^1-32^ double filaments on lipid monolayers showed that EcFtsA-FtsN^1-32^ double filaments have an architecture very similar to the VmFtsA^1-396^ double filaments determined by crystallography (PDB 7Q6F) (Figure 2f). To corroborate, we designed a lateral interface mutant of *E. coli* FtsA based on the VmFtsA double filament structure: EcFtsA^M96E, R153D^ (Figure 2g, right). The lipid monolayer assay showed that EcFtsA^M96E, R153D^ was indeed deficient in EcFtsN^1-32^-dependent double filament formation (Figure 2g).

The short cytoplasmic tail of FtsN harbours two sequence motifs that are conserved among *E. coli* and related proteobacteria with similar FtsA and FtsN sequences: a conserved ^3^R/KDY^6^ (*E. coli* amino acid positions) motif near the N-terminus^23^ and two to three stretches of basic amino acids, the most prominent of which in *E. coli* FtsN is ^16^RRKK^19^ ^22^ (Extended Data Figure 2a, g). In *E. coli*, a D5N mutation in the conserved N-terminal motif of FtsN was shown to impair FtsA-FtsN interactions *in vivo*^23^, but did not affect binding or co-localisation with FtsA-FtsZ filaments on supported lipid bilayers *in vitro*^24^. In contrast, and somewhat confusingly, mutation of the basic ^16^RRKK^19^ stretch abrogated binding *in vitro*^22,24^, yet was permissible *in vivo* under conditions for which the FtsA-FtsN interaction becomes essential^23^. We therefore tested a set of EcFtsN^1-32^ truncations and mutations in our SPR and monolayer assays to determine the effect of those modifications on the FtsA-FtsN interaction and on double filament formation (Extended Data Figure 2h, i). Mutation or deletion of any one of the three stretches of basic amino acids in EcFtsN^1-32^ reduced binding affinity for FtsA and, consequently, its ability to promote FtsA double filament formation. EcFtsN^1-32, D5N^ and EcFtsN^1-33, scrambled^ (Supplementary Table T5) did not show reduced binding affinity, but were unable to induce FtsA double filament formation. Hence, the D5N mutation in FtsN might be non-permissible *in vivo* under conditions for which the FtsA-FtsN interaction becomes essential because it fails to promote formation of FtsA double filaments. In other words, this result supports the idea that FtsA double filaments are important for the activation or correct regulation of cell division by FtsA and FtsN.

We then tested whether any of the FtsN suppressor^16,27^ or filament architecture and ZipA suppressor mutations^32^ in FtsA (mutants summarised in Supplementary Table T2) would impact FtsN-induced double filament formation of FtsA (Figure 1c). While the FtsN suppressor mutants FtsA^E124A^ and FtsA^I143L^, similar to wildtype FtsA, formed “mini-rings” in the absence of EcFtsN^1-32^, they more readily formed double filaments than wildtype FtsA at three- (7 μM) and six-fold (13 μM) molar excess of EcFtsN^1-32^ (Figure 2a). The filament architecture and ZipA suppressor mutants FtsA^G50E^ and FtsA^R286W^ formed fewer “mini-rings” than wildtype FtsA in the absence of FtsN (Figure 2a). For FtsA^G50E^, “mini-rings” and short double filaments were observed. FtsA^R286W^ predominantly formed arcs, curved, short and single filaments. Similar to the FtsA^E124A^ and FtsA^I143L^ proteins, FtsA^G50E^ and FtsA^R286W^ formed many more double filaments than wildtype FtsA at three- (7 μM) and six-fold (13 μM) molar excess of EcFtsN^1-32^ (Figure 2a). Thus, both FtsN suppressor as well as ZipA suppressor and FtsA filament architecture mutants appear to enhance FtsN-dependent FtsA double filament formation.

### VmFtsN^1-29^ binds VmFtsA^1-396^ in the IA-IC interdomain cleft

**Extended Data Figure 3:**
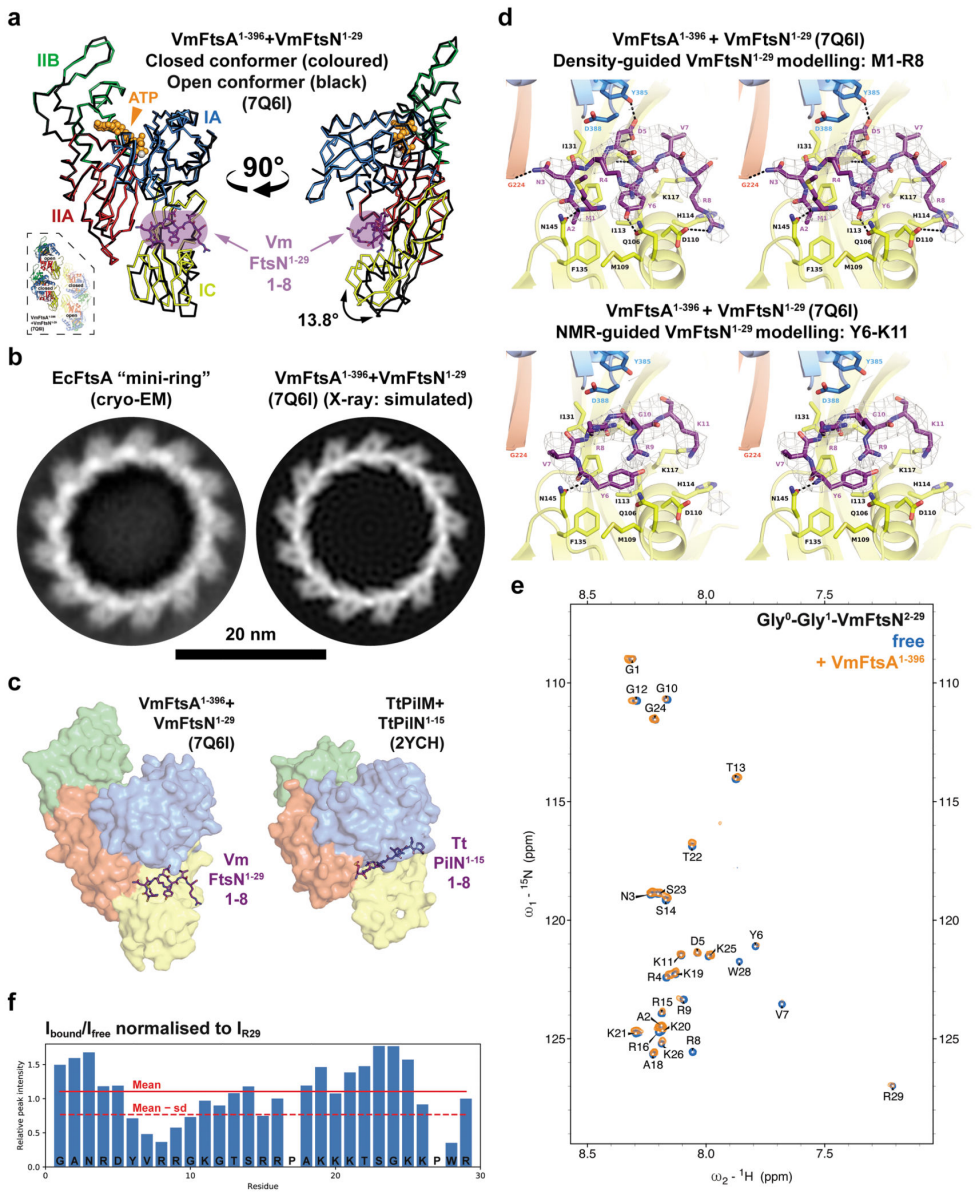
Modelling of VmFtsN^1-29^ binding to VmFtsA^1-396^. **a**, Comparison between the peptide-bound closed (coloured) and peptide-free open conformer (black) of FtsA in the VmFtsA^1-396^-VmFtsN^1-29^ co-crystal structure (PDB 7Q6I). The IC domain of the open conformer is rotated 13.8° downwards compared to the closed conformer, as determined by analysis with DynDom^38^. Consequently, the open conformation is likely incompatible with VmFtsN^1-29^ binding. The inset shows the position of open and closed conformers within the tetramer. **b**, Left: EcFtsA forms “mini-rings” on lipid monolayers as determined by cryo EM. Two independent grids were examined. Data was collected on one grid. Right: a computed 2D projection after expansion of a longitudinal dimer from the VmFtsA^1-396^-VmFtsN^1-29^ co-crystal structure (PDB 7Q6I) is shown for comparison. The expanded longitudinal dimer does not form a closed ring but a helix. The comparison illustrates that FtsA’s IA domains are facing outwards. Scale bar, 20 nm. **c**, Comparison between the *V. maritimus* FtsA-FtsN and *Thermus thermophilus* PilM-PilN (PDB 2YCH) interaction sites^39^. Both binding sites are in the IA-IC interdomain cleft of FtsA and PilM but occupy distinct subspaces. FtsN predominantly contacts the IC domain of FtsA, whereas PilN binds closer to the IA domain of PilM. **d**, Stereo images of the FtsA-FtsN interaction site in the VmFtsA^1-396^-VmFtsN^1-29^ co-crystal structure (PDB 7Q6I). Top: our preferred interpretation of the electron density corresponding to VmFtsN^1-29^, with residues M1-R8 modelled (purple). Side chains of FtsA residues in the interaction site are shown as sticks and polar contacts are marked with black, dashed lines. Bottom: electron density interpretation guided by the NMR data instead (panels e and f), with residues Y6-K11 of VmFtsN^1-29^ modelled (purple). Electron density maps (grey) are shown at 1.2 sigma. **e**, ^1^H, ^15^N 2D-HSQC NMR spectrum of free Gly-Gly-VmFtsN^2-29^ (blue) and with equimolar amounts of FtsA added (orange). To follow VmFtsN numbering, the first glycine of Gly-Gly-VmFtsN^2-29^ is assigned as G0. **f**, Changes in relative peak intensity expressed as I_bound_/I_free_ with intensities normalised to I_R29_, which is assumed not to be involved in the VmFtsA-FtsN interaction.

**Figure 3:**
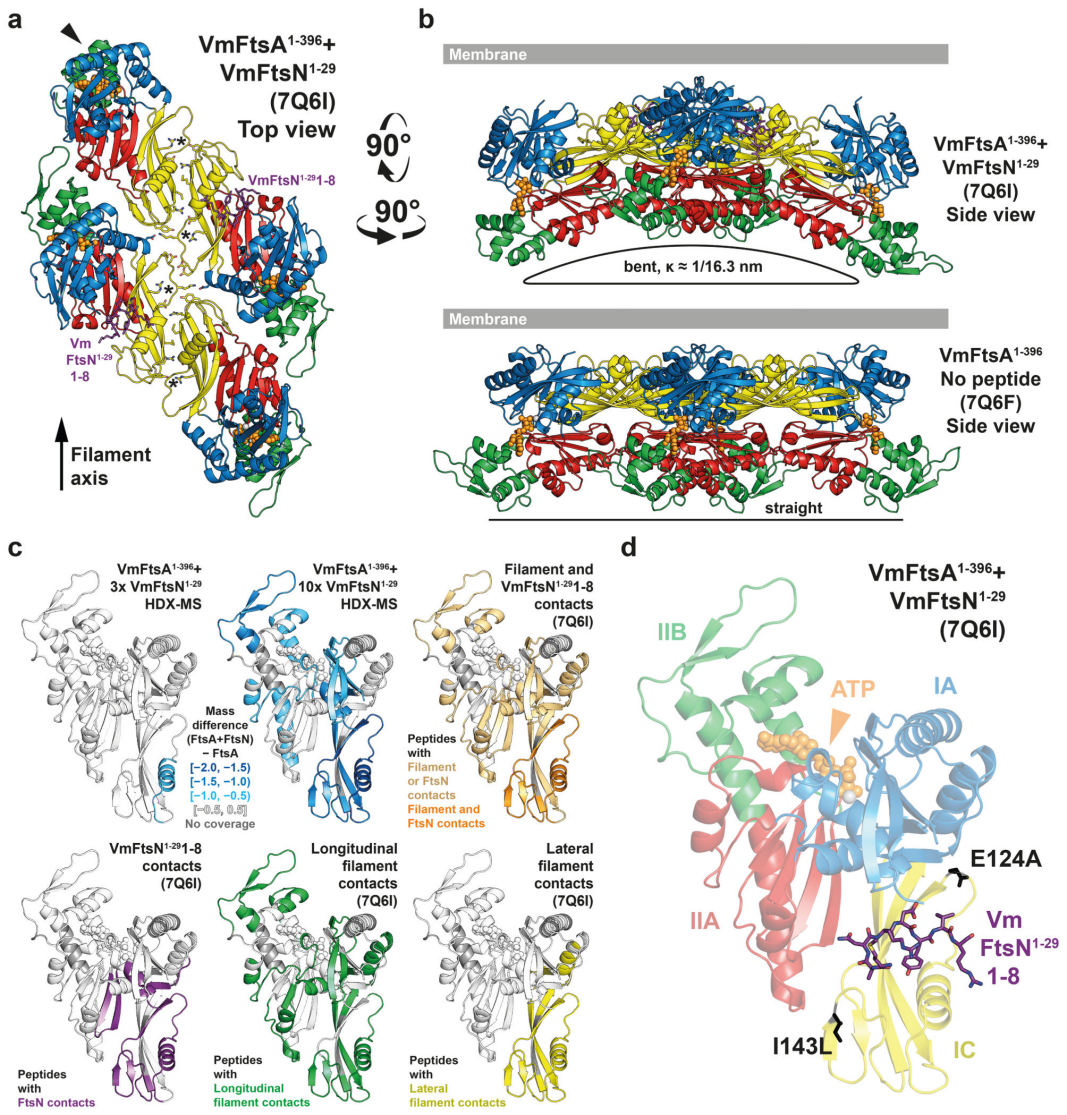
VmFtsA^1-396^ and VmFtsN^1-29^ crystallise as short, bent double filaments. **a**, 16 FtsA monomers in the ASU of the co-crystal structure are organised into short, antiparallel, and curved tetramers (see also Supplementary Movie M1). Top view of a representative tetramer (which requires crystal symmetry to be applied to the PDB coordinates) from the membrane-proximal side. Residues 1-8 of VmFtsN^1-29^ (purple sticks) are present in the closed subunit of each protofilament. IC domains are tilted against each other compared to the VmFtsA^1-396^ double filament structure (PDB 7Q6F), presumably destabilising the R91-Y139 interaction (asterisks) in the lateral interface. The IIB domain of the top left monomer (chain D) is partially disordered (arrowhead). **b**, A comparison of the side views of the PDB 7Q6I and PDB 7Q6F crystal structures illustrates that the 7Q6I tetramer is bent along the filament axis. **c**, HDX-MS analysis of VmFtsA^1-396^ with three- (30 μM) or ten-fold molar excess (100 μM) of VmFtsN^1-29^, confirming VmFtsN^1-29^ to bind in the IA-IC interdomain cleft of FtsA. Peptides that are protected in the presence of VmFtsN^1-29^ (slower exchange of hydrogen for deuterium) are highlighted in blue. Presumably because high concentrations of VmFtsN^1-29^ induce polymerisation of VmFtsA^1-396^ (Extended Data Figure 2d, e), peptides mapping to the filament interfaces are protected as well. For orientation, peptides are coloured according to their involvement in different interfaces of the VmFtsA^1-396^-VmFtsN^1-29^ co-polymer (PDB 7Q6I). **d**, Residues 1-8 of VmFtsN^1-29^ bind in the IA-IC interdomain cleft of VmFtsA^1-396^. Positions of FtsN suppressor mutations E124A27 and I143L16 are shown in black.

To understand the FtsA-FtsN interaction at the molecular level, we co-crystallised VmFtsA^1-396^ with the *V. maritimus* cytoplasmic FtsN peptide, VmFtsN^1-29^. The VmFtsA^1-396^-VmFtsN^1-29^ co-crystal structure (PDB 7Q6I) showed a larger unit cell than that obtained for the VmFtsA^1-396^ double filament structure [PDB 7Q6F, two FtsA monomers per asymmetric unit (ASU)], encompassing 16 FtsA monomers per ASU (Supplementary Table T1). The 16 FtsA monomers are organised into four tetramers, each containing two short antiparallel protofilaments comprising two FtsA monomers (Figure 3a; Supplementary Movie M1). One of the two FtsA monomers in each protofilament adopts a closed and the other one an open conformation (Extended Data Figure 3a). The positions of the IC domains in the closed (PDB 7Q6I chain A) and open (PDB 7Q6I chain B) conformation are similar but not identical to the IC domain position in the VmFtsA^1-396^ double filament (PDB 7Q6F) (Extended Data Figure 1b). Only in the IA-IC interdomain cleft of closed FtsA monomers could we observe electron density for the VmFtsN^1-29^ peptide (Figure 3a, d; Extended Data Figure 3a). The IC domain in the open conformation is rotated downwards by 13.8 ° compared to the closed conformation, as analysed by DynDom^38^ (Extended Data Figure 3a). This means the open conformation is likely incompatible with VmFtsN^1-29^ binding (Extended Data Figure 3a). The IC domains in the lateral interfaces of each tetramer are twisted against each other compared to the straight VmFtsA^1-396^ double filament (Figure 3a). Several hydrogen bonding contacts are lost because of the bending when compared to the straight filament (Figure 3a, asterisks; Figure 3b). The VmFtsA^1-396^-VmFtsN^1-29^ tetramer is bent along the filament axis with an estimated radius of curvature of 16.3 nm (Figure 3b). We noticed that symmetry expansion of a longitudinal dimer present in the VmFtsA^1-396^-VmFtsN^1-29^ tetramer results in a helix of ~20 nm diameter, matching the diameter reported for FtsA “mini-rings”^37^. A comparison between the atomic model of the symmetry-expanded helix and a cryo-EM 2D class average of EcFtsA “mini-rings” assembled on lipid monolayers shows that FtsA’s IA domain is facing outwards in the “mini-rings” (Extended Data Figure 3b). In contrast to a previous study^37^, ~65 % of intact “mini-rings” we observed were comprised of 13 FtsA monomers, whereas 35 % were 12-subunit “mini-rings”. Presumably because of their favourable rotational symmetry, 12-subunit “mini-rings” were mostly organised into 2D arrays as reported by Krupka and co-workers.

Due to the low resolution (3.6 Å) of the VmFtsA^1-396^-VmFtsN^1-29^ co-crystal structure (Supplementary Table T1), we could not unambiguously interpret the electron density map representing about seven to eight amino acids of the VmFtsN^1-29^ peptide. We therefore confirmed the location of the VmFtsN^1-29^ binding site in VmFtsA^1-396^ by hydrogen deuterium exchange mass spectrometry (HDX-MS) (Figure 3c). At three-fold molar excess (30 μM) of VmFtsN^1-29^ over VmFtsA^1-396^, only helix 2 located in the IC domain of FtsA showed significantly reduced deuterium incorporation (lower mass differential) compared to free VmFtsA^1-396^ (Figure 3c, top left). This agrees with VmFtsN^1-29^ binding across the IC domain as seen in the co-crystal structure (Figure 3d). At 10-fold molar excess (100 μM) of VmFtsN^1-29^, FtsA peptides in filament interfaces were also protected, again, most likely indicating that binding of the cytoplasmic FtsN peptide to FtsA facilitates filament formation (Figure 3c).

As proposed previously^22^, the FtsA-FtsN interaction is reminiscent of that between PilM and PilN, two proteins involved in type IV pilus formation^39^. PilM, which also has a IC subdomain, is structurally related to FtsA, and PilN, similar to FtsN, is a bitopic membrane protein with a short, cytoplasmic tail comprising about 30 amino acids^39^. FtsN and PilN both bind in the IA-IC interdomain cleft (using FtsA domain nomenclature for PilM), but use different binding modes (Extended Data Figure 3c). PilN makes extensive contacts with the IA domain of PilM. FtsN binds further downward in the cleft, predominantly contacting the IC domain of FtsA.

To try to assign the VmFtsN^1-29^ peptide sequence in the VmFtsA^1-396^-VmFtsN^1-29^ co-crystal structure (PDB 7Q6I) we reasoned that based on the geometry *in vivo*, where FtsA binds to the cell membrane through its C-terminal amphipathic helix, the N-terminal half of VmFtsN^1-29^ is expected to interact with FtsA. The globular domain of FtsA was reported to be several nanometres away from the inner membrane in *E. coli*^40^. The C-terminal half of the VmFtsN^1-29^ peptide would be confined to the proximity of the inner membrane, as it is linked to the transmembrane helix in the full-length FtsN protein (Extended Data Figure 2a). Amino acids M1-R8 of VmFtsN^1-29^ provided the best fit for the density with reasonable chemistry, i.e. several hydrogen bonds to FtsA and a hydrophobic pocket accommodating the central tyrosine (Y6) in the sequence stretch (Extended Data Figure 3d, top). This model also implies a central position of aspartate D5 within the FtsA-FtsN interaction site that could point to a mechanism by which the D5N mutation impairs double filament formation (Extended Data Figure 2h; Extended Data Figure 3d, top). In that case, FtsN^D5N^ would not be able to bridge FtsA’s IA and IC domain. We also studied the VmFtsA^1-396^-VmFtsN^1-29^ interaction by nuclear magnetic resonance (NMR) spectroscopy. We assigned the peptide backbone of Gly-Gly-VmFtsN^2-29^, a VmFtsN^1-29^ construct in which two glycine residues replace the N-terminal methionine (Extended Data Figure 3e). Next, we analysed peak broadening (reduction in peak intensity due to the slower tumbling rate of FtsA-bound Gly-Gly-VmFtsN^2-29^ peptide) upon addition of equimolar amounts of VmFtsA^1-396^ (Extended Data Figure 3f). Intensity reduction was most prominent in the stretch of amino acids Y6-G10 (Extended Data Figure 3f), suggesting a slightly shifted binding motif compared to our density-based assignment (M1-R8) (Extended Data Figure 3d). We were not able to generate a good fit of these residues into the electron density map of the VmFtsA^1-396^-VmFtsN^1-29^ co-crystal structure. For technical reasons, the NMR experiments were carried out at pH 6.0, whereas binding experiments were done at pH 7.7 and crystallisation was achieved at pH 8.5.

### FtsA double filaments adopt negative Gaussian curvature

Based on the observation that VmFtsA^1-396^-VmFtsN^1-29^ double filaments were curved in the co-crystal structure (Figure 3b), we hypothesised that the intrinsic curvature-preference observed for MreB double filaments might also be a feature of FtsA-FtsN double filaments^4,34^. The curvature preference of MreB filaments has been proposed to enable a curvature-sensing mechanism that allows them to robustly align in cells with the axis of highest principal curvature, which corresponds to the short axis of rod-shaped bacteria such as *E. coli* and *B. subtilis*^34,35^.

**Figure 4:**
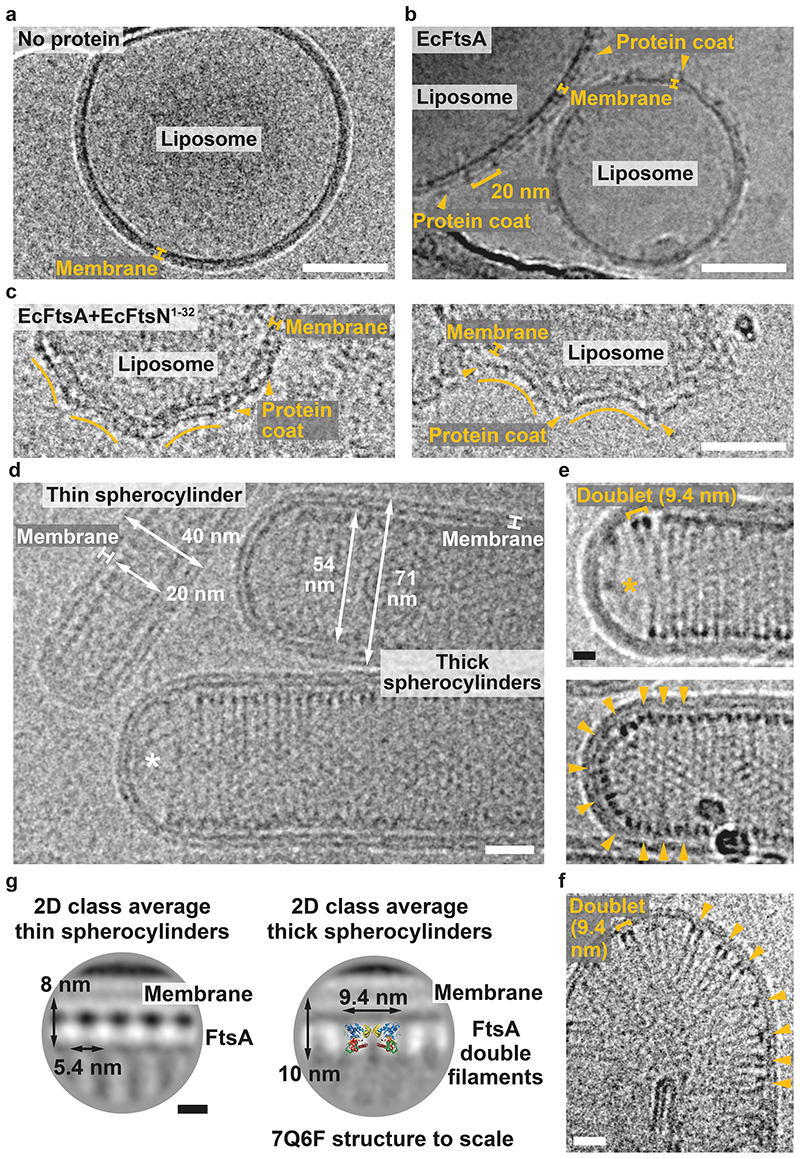
EcFtsA-FtsN^1-32^ double filaments orient inside liposomes. **a**, Cryo-EM micrograph showing a spherical liposome with no proteins added. The carbon edge of the grid is visible at the top of the image. Scale bar, 50 nm. **b**, Cryo-EM micrograph of liposomes with EcFtsA (arrowheads) bound to the outside. Liposomes show no indentations. The protein coat shows a 20 nm repeat, indicating the presence of EcFtsA “mini-rings” (see also Extended Data Figure 3b). The carbon edge of the grid is visible at the bottom left of the image. Scale bar, 50 nm. **c**, Cryo-EM micrographs of liposomes with EcFtsA-FtsN^1-32^ filaments (arrowheads) bound to the outside. Liposomes show indentations. See Salje *et al*. for a comparison with MreB filaments added to liposomes4. Scale bar, 50 nm. **d**, Co-encapsulation of EcFtsA and EcFtsN^1-32^ in liposomes produces thin and thick protein-filled spherocylinders (rods) with diameters of ~40 nm and ~70 nm, respectively. EcFtsA-FtsN^1-32^ filaments align with the short axis of the spherocylinders. There are no filaments in the hemisphere of a thick spherocylinder (asterisk). See Hussain *et al*. for a comparison with MreB filaments encapsulated into liposomes^34^. Scale bar, 20 nm. **e**, Cryo-EM micrographs of EcFtsA-FtsN^1-32^ double filaments in thick spherocylinders. Top: EcFtsA-FtsN^1-32^ double filaments align with the short axis of thick spherocylinders. There are no filaments in the hemisphere of the spherocylinder (asterisk). Bottom: EcFtsA-FtsN^1-32^ double filaments align with the short axis in the cylindrical part of the spherocylinder but are randomly oriented in the hemisphere (arrowheads). Scale bar, 10 nm. **f**, Liposome with few EcFtsA-FtsN^1-32^ double filaments inside. EcFtsA-FtsN^1-32^ double filaments are randomly oriented in the semi-circular part of the liposome but are more aligned in the cylindrical part (arrowheads). Scale bar, 20 nm. For panels a-f, at least two independently prepared grids were examined per condition. **g**, 2D class averages of FtsA membrane attachment sites in thin and thick spherocylinders. EcFtsA-FtsN^1-32^ are organised into single protofilaments in thin spherocylinders. Thick spherocylinders contain EcFtsA-FtsN^1-32^ double filaments. End view of the VmFtsA^1-396^ double filament structure (PDB 7Q6F) shown to scale. Scale bar, 5 nm.

To investigate the curvature preference of FtsA filaments, we added EcFtsA-FtsN^1-32^ to preformed liposomes made from *E. coli* polar lipid extract. In the absence of proteins liposomes are typically round or oval (Figure 4a). Liposomes coated with EcFtsA alone are also round or oval (Figure 4b). The FtsA coat shows a 20 nm repeat indicating the presence of FtsA “mini-rings”^37^ (see also Extended Data Figure 3b). In contrast, liposomes coated with EcFtsA-FtsN^1-32^ double filaments showed membrane indentations with negative Gaussian curvature (Figure 4c). We encourage the reader to revisit previously published images of MreB double filaments bound to the outside of liposomes showing a remarkably similar binding mode^4,33^. To better match the geometry inside an *E. coli* cell, we then encapsulated EcFtsA-FtsN^1-32^ double filaments in liposomes. Protein-filled liposomes were spherocylindrical (rod-shaped) or had spherocylindrical protrusions (Figure 4d). Spherocylinders were either ~40 nm or ~70 nm in diameter. Thicker spherocylinders contained tightly packed EcFtsA-FtsN^1-32^ double filaments that were aligned with the short axis in the cylindrical section (Figure 4d, e). We again suggest the reader to compare with published images of MreB filaments inside liposomes showing similar filament arrangements^34^. EcFtsA-FtsN^1-32^ filaments were regularly absent in the hemispheres (poles) of thick spherocylinders (Figure 4d, e). If filaments were present in the hemispheres, they showed random angular orientations (Figure 4e, bottom). Rarely, we obtained liposomes with only a few EcFtsA-FtsN^1-32^ double filaments inside (Figure 4f). Again, filaments were distributed randomly in spherical sections of the liposomes but aligned with the short axis of the liposomes in more cylindrical sections. We performed 2D averaging and image classification focussed on the membrane attachment sites of the FtsA filaments in thin and thick spherocylinders. Class averages clearly illustrated the different organisation of FtsA filaments in thin and thick spherocylinders (Figure 4g). In thin spherocylinders, FtsA was organised into bent single protofilaments (Figure 4g, left). In thick spherocylinders however, FtsA formed bent double filaments, as apparent by superposition of the VmFtsA^1-396^ double filament crystal structure (PDB 7Q6F) onto the 2D class average (Figure 4g, right).

### *E. coli* FtsA forms antiparallel double filaments *in vivo*

**Extended Data Figure 4:**
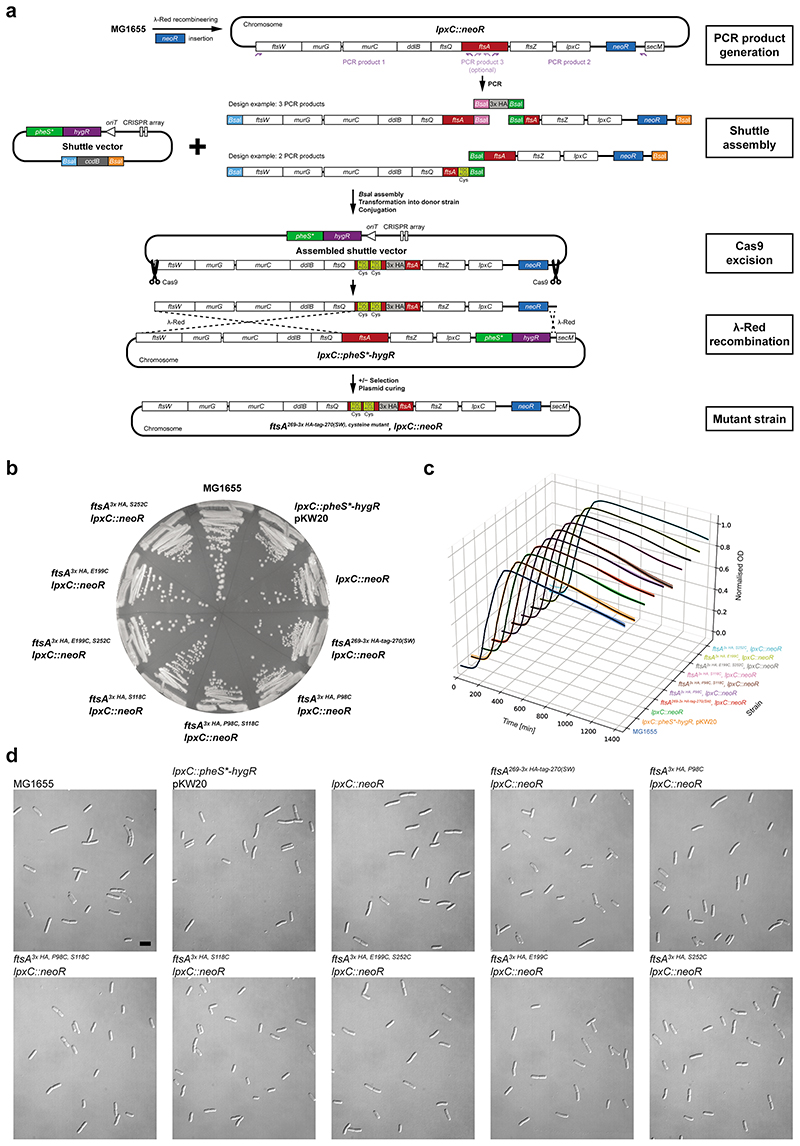
FtsA cysteine mutant strains: generation and absence of phenotype changes. **a**, Workflow for REXER^72^-based strain construction. PCR products containing the 3x HA-tag or cysteine point mutations were inserted into a shuttle vector by Golden Gate assembly^77^. Assembled shuttle vectors were transformed into the donor strain, conjugated, and excised *in vivo* using Cas9. Targeting constructs contain homology regions for λ Red mediated recombination into the target locus. Recombinants were selected for *neoR* and *tetR* markers and against the pheS* marker. Strains were cured of the helper plasmid pKW20 by growth in absence of selection. **b**, Growth of strains containing single or double cysteine point mutations and a 3x HA-tag in the endogenous *ftsA* gene, and a kanamycin resistance cassette inserted after the *lpxC* gene. Parent strains and the original MG1655 strain are also shown. SW: sandwich fusion. **c**, Growth curves of the same strains in liquid LB medium. Plotted are traces of technical octuplicates (coloured) with mean (black). **d**, DIC images of the same strains in exponential phase (OD_600_ = 0.2-0.3) demonstrating the absence of elongated cells. Similar results were achieved in biological triplicate, of which one was imaged using DIC and two were imaged using phase contrast.

**Extended Data Figure 5:**
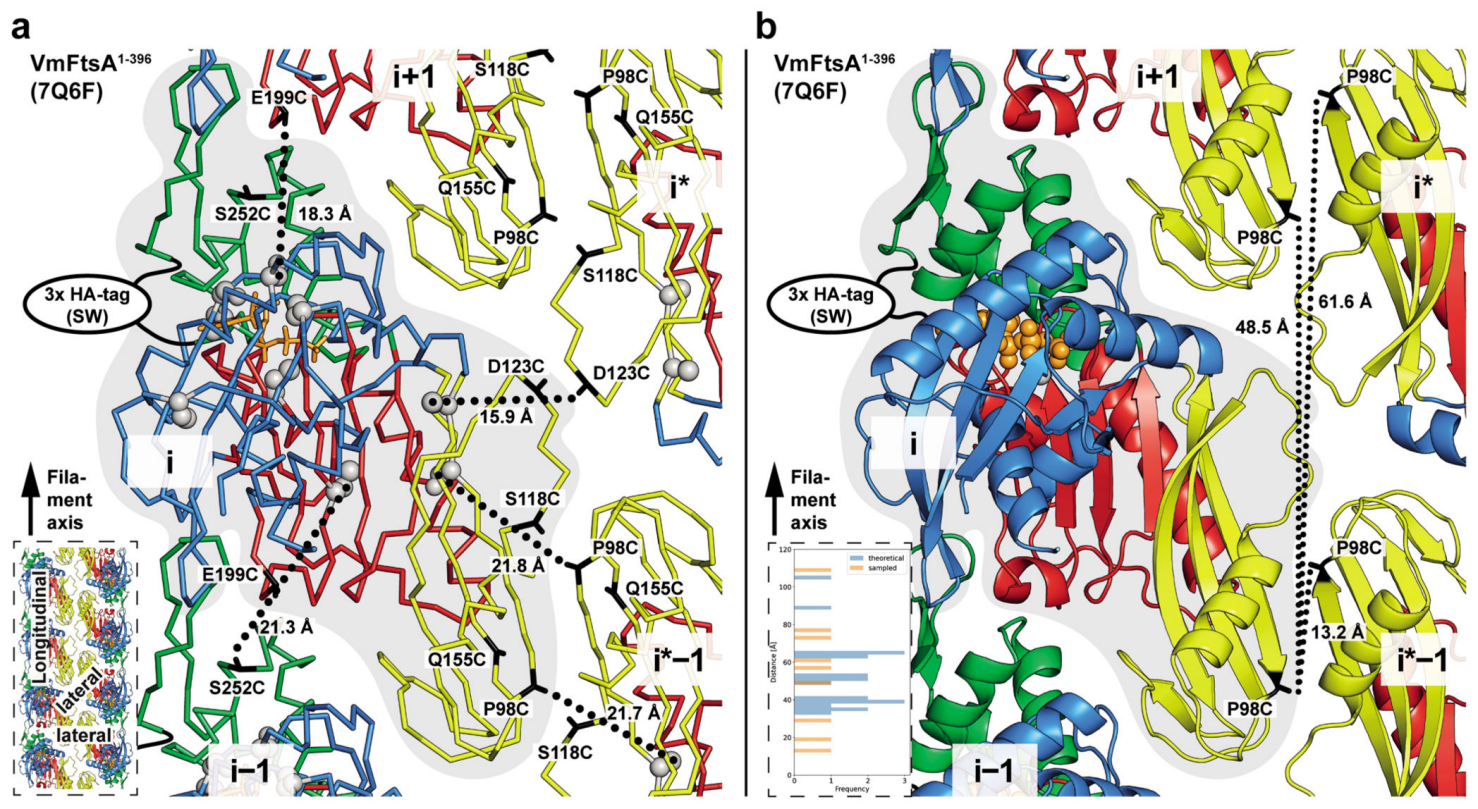
Distances between endogenous and mutated cysteines in the FtsA double filament. **a**, Positions of endogenous cysteines in EcFtsA (grey spheres) and of all cysteine mutations used for *in vivo* cysteine cross-linking (black sticks) (Figure 5a) are highlighted on the VmFtsA double filament structure (PDB 7Q6F). Dotted lines indicate intermolecular C_β_-C_β_ (putative cross-link) distances between selected cysteine mutations and the closest endogenous cysteine, the shortest distance being 15.9 Å (FtsA_i_ C163-FtsA_i*_ D123C). The inset highlights the interfaces in the VmFtsA double filament. SW: sandwich fusion. **b**, Single cysteine point mutations serve as controls for distance-independent intermolecular cross-linking because of the symmetry of the FtsA double filament, as illustrated on the example of P98C. The P98C mutation used for *in vivo* cysteine cross-linking is highlighted on the VmFtsA double filament structure (PDB 7Q6F). C_β_-C_β_ distances between intermolecular P98C mutations are indicated by dotted lines. The inset provides a comparison between experimentally sampled intermolecular C_β_-C_β_ distances by single cysteine point mutations (orange) and all intermolecular C_β_-C_β_ distances between amino acids P98, S118, E199 and S252 (blue). Single cysteine point mutations sample intermolecular distances similar to those between double cysteine mutations. Calculated intermolecular C_β_-C_β_ distances were rounded to one digit and duplicate values removed prior to plotting.

**Figure 5:**
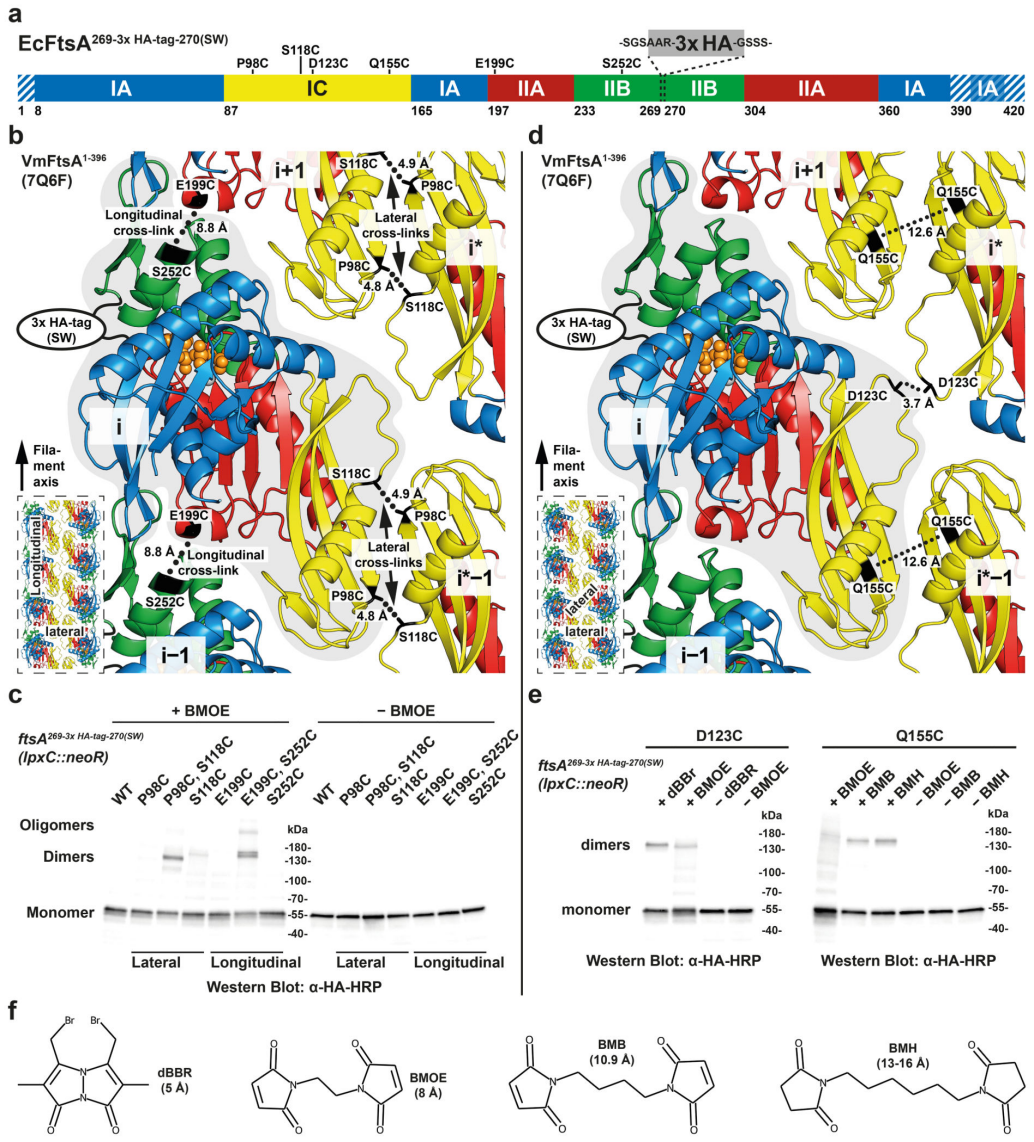
FtsA forms antiparallel double filaments *in vivo*. **a**, Domain architecture of EcFtsA (amino acids positions are the same for VmFtsA). Positions of cysteine mutations and the 3x HA-tag (comprising 40 amino acids including linkers, grey) are indicated. Striped areas correspond to disordered residues in the VmFtsA^1-396^ double filament structure (PDB 7Q6F). **b**, Cysteine mutation pairs FtsA^3x HA, P98C, S118C^ and FtsA^3x HA, E199C, S252C^ probing the lateral and longitudinal filament interfaces, respectively, are highlighted on the VmFtsA double filament structure (PDB 7Q6F). C_β_-C_β_ distances between cysteines and the position of the 3x HA-tag are indicated. The inset highlights the probed interfaces in the context of the double filament. SW: sandwich fusion. **c**, Western blot of cell lysate from FtsA cysteine mutant *E. coli* strains after *in vivo* cysteine cross-linking (+ BMOE) or without cross-linking (− BMOE). Signal for covalent FtsA dimers can be detected for both double cysteine mutants. For *ftsA^3x HA, E199C, S252C^*, higher order oligomers can be detected because of the open symmetry of the longitudinal contact, which leads to chaining. Similar results were achieved in biological triplicate. **d**, Cysteine mutations *ftsA^3x HA, D123C^* and *ftsA^3x HA, Q155C^* probing the lateral FtsA_i_-FtsA_i*_ and FtsA_i_-FtsA_i*−1_ filament interfaces, respectively, are highlighted on the VmFtsA double filament structure (PDB 7Q6F). C_β_-C_β_ distances between cysteines and the position of the 3x HA-tag are indicated. Both single cysteine mutations utilise the local C2 symmetry of the respective lateral interface for cross-linking. The inset highlights the probed interfaces in the context of the double filament. **e**, Western blot of cell lysate from FtsA cysteine mutant *E. coli* strains after *in vivo* cysteine cross-linking with thiol-directed cross-linkers of different lengths (“+ cross-linker”) or without cross-linking (“− cross-linker”). Signal for a FtsA dimer can be detected after using dBBr and, to a lesser extent, BMOE cross-linking in the *ftsA^3x HA, D123C^* mutant. For the *ftsA^3x HA, Q155C^* mutation, FtsA dimers can be detected after cross-linking with BMB or BMH, but not with BMOE. Similar results were achieved in biological duplicate. **f**, Structures and estimated cross-linking distances for cross-linkers used in this study. dBBr: dibromobimane, BMOE: bismaleimidoethane, BMB: 1,4-bismaleimidobutane, BMH: bismaleimidohexane.

To validate our *in vitro* findings, we investigated double filament formation of FtsA *in vivo* using site-specific cysteine cross-linking in *E. coli*. We inserted a *neoR* marker downstream of *lpxC* for positive selection (Extended Data Figure 4a). For visualisation via Western blotting, we introduced a 3x HA-tag (comprising 40 amino acids including linkers) into the H7-S12 loop of FtsA^41^. Cysteine mutations for *in vivo* site-specific cross-linking were designed based on the VmFtsA^1-396^ double filament crystal structure (PDB 7Q6F) (Figure 5a; see also Extended Data Figure 5a). Mutant strains showed no growth defects and no elongated cells compared to the MG1655 parent strain (Extended Data Figure 4b-d).

This way we generated FtsA double cysteine mutant strains *ftsA^3x^ ^HA,^ ^P98C,^ ^S118C^* and *ftsA^3x HA, E199C, S252C^*, which probe the lateral FtsA_i_-FtsA_i*−1_ and the longitudinal FtsA_i_-FtsA_i±1_ interface of the FtsA double filament, respectively (Figure 5b). *E. coli* cells were treated with bismaleimidoethane (BMOE) in exponential phase (OD_600_ = 0.2-0.4). BMOE enters living *E. coli* cells and rapidly cross-links closely spaced thiols such as cysteine side chains *in vivo*. FtsA species were visualised by Western blotting against the 3x HA-tag in FtsA (Figure 5c). We detected efficient formation of crosslinked FtsA dimers for both the *ftsA^3x HA, P98C, S118C^* and *ftsA^3x HA, E199C, S252C^* double mutants, but only weak background signal spread across multiple species in single cysteine mutant controls (Figure 5c; see also Extended Data Figure 5a). We also detected higher order polymers for the *ftsA^3x HA, E199C, S252C^* mutant, presumably because the open symmetry of the longitudinal filament contact allows for more than two FtsA monomers to be cross-linked through chaining.

To probe the lateral association further, we generated the two, additional FtsA single cysteine mutant strains *ftsA^3x HA, D123C^* and *ftsA^3x HA, Q155C^*, which probe the lateral FtsA_i_-FtsA_i*_ and FtsA_i_-FtsA_i*−1_ interface of the FtsA double filament, respectively (Figure 5d). These single cysteine mutants may cross-link to their symmetry mates because of the local C2 symmetry in each of the two lateral filament interfaces. However, C_β_-C_β_ distances in the VmFtsA double filament structure of 3.7 Å for FtsA^3x HA, D123C^ and 12.6 Å for FtsA^3x HA, Q155C^ indicated that cross-linking with BMOE with an expected cross-linking distance of ~8 Å might be inefficient, which prompted us to try thiol-directed cross-linkers of different lengths (Figure 5f). The *ftsA^3x^ ^HA, D123C^* mutant showed more efficient cross-linking of FtsA using dibromobimane (dBBr) than using BMOE (Figure 5e, left). In case of the *ftsA^3x HA, Q155C^* mutant, BMOE cross-linking did not lead to efficient formation of covalent FtsA dimers, whereas treatment with the longer maleimide cross-linkers 1,4-bismaleimidobutane (BMB) and bismaleimidohexane (BMH) did (Figure 5e, right). Taken together, our data strongly suggest that FtsA forms protofilaments in cells and, that these protofilaments are further arranged into antiparallel double filaments as suggested by the VmFtsA^1-396^ crystal structure (PDB 7Q6F).

## Conclusions

**Figure 6:**
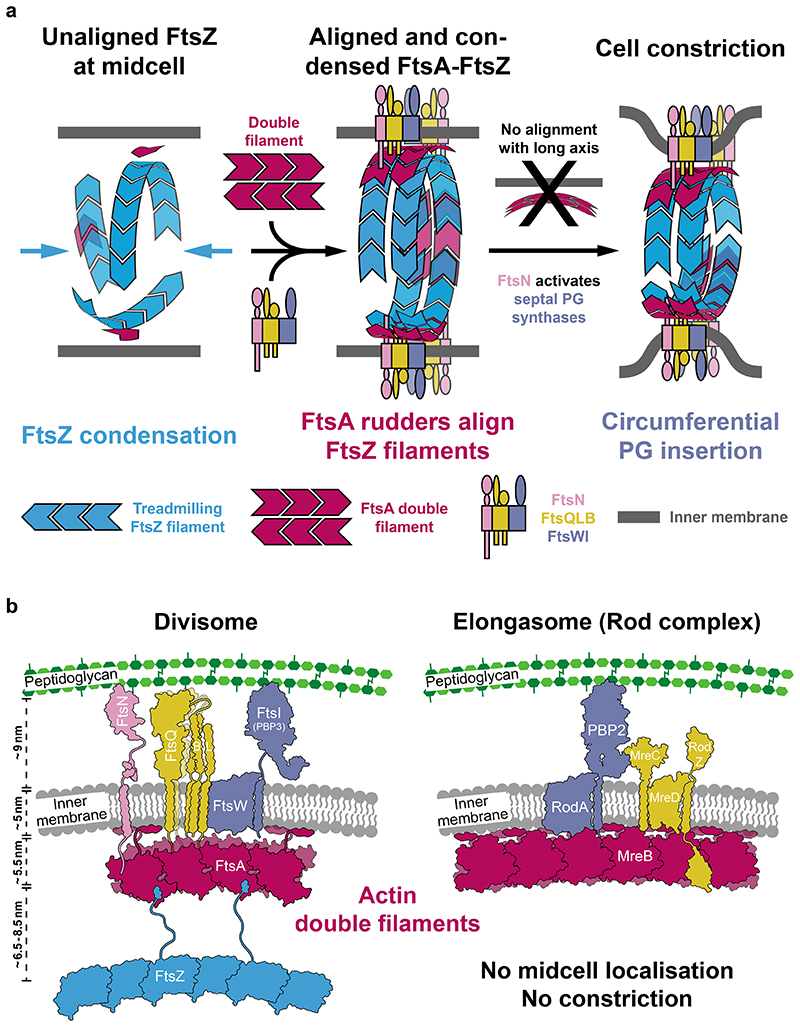
Model septal PG synthesis and cell constriction. **a**, Logical steps towards a divisome primed for curvature-guided septal PG synthesis. The temporal order of events remains to be determined. FtsZ filaments recruited to midcell are unaligned (left). Downstream divisome proteins are recruited and condensed into the narrow midcell plane by treadmilling FtsZ filaments that themselves will also be aligned through their interaction with curvature-sensing FtsA filaments (middle). Condensed complexes are aligned with the short axis of the cell by FtsA double filaments because of their curvature-sensing mechanism, which we propose here they share with MreB. Finally, FtsZ filaments distribute divisome components via treadmilling and might thereby reinforce their alignment with the short axis of the cell. FtsN is required for FtsA double filament formation and activation of septal PG synthases. By coordinating both activities across the inner membrane, FtsN might constitute a synchronising activation switch for the divisome, which allows circumferential synthesis of septal peptidoglycan through the alignment activity, and at the same time cell constriction to commence through the activation of PG synthesis (right). **b**, Schematic overview of core components of the divisome and elongasome highlighting the evolutionary relationship between the two complexes. We propose here that both complexes utilise the curvature-sensing properties of their cytoplasmic actin double filament scaffolds (red) to direct peptidoglycan synthesis around the cell’s circumference. The bipartite PG synthases (FtsWI and RodA-PBP2, purple) are connected to the actin double filaments via integral membrane proteins that serve as structural and regulatory subunits (yellow), or potentially even directly. Unlike the elongasome, the divisome, in FtsN, possesses an additional regulatory subunit that might be necessary since cell division is regulated during the cell cycle. FtsZ is absent in the elongasome. FtsZ localises the divisome and its activities to midcell and probably more importantly into a narrow plane, an activity that is neither required nor desired in the elongasome.

We report that FtsA polymerises to form antiparallel double filaments in *E. coli* and find that filament formation is induced through binding to the cytoplasmic tail of FtsN *in vitro*. The only other actin-like protein known to polymerise into antiparallel double filaments is MreB, the actin homologue in the elongasome, which serves as a “rudder” to guide peptidoglycan insertion in the cell wall during growth^33,34,31^ We observed that FtsA-FtsN filaments preferentially bind surfaces of negative Gaussian curvature in and on liposomes, as do MreB double filaments^4,33,34^ (Figure 4). We therefore propose that MreB and FtsA have a common curvature sensing mechanism. Finally, we devised a model for curvature-guided cell constriction by FtsA-FtsN double filaments, which align the direction of the divisome’s glycan strand synthesis activity with the circumference of the cell (Figure 6a).

In our model, we define three phases in divisome assembly and maturation. In phase 1, unaligned FtsZ filaments are present at midcell, in phase 2 a fully assembled divisome aligns with the short axis of the cell, and in phase 3 the divisome is fully activated, synthesising septal PG and enabling cell constriction (Figure 6a). We note that the temporal order of individual recruitment and activation events during divisome maturation remains largely unknown, and could be informed by *in vivo* single molecule imaging of FtsA. FtsZ filaments are present at midcell, presumably to determine the division plane, but are unaligned in the absence of an alignment mechanism (Figure 6a, left). After recruitment of divisome proteins, and Z-ring condensation^10,11^, which has previously been proposed to be driven by a FtsA “mini-ring” to double filament transition^32,37^, FtsN-induced FtsA double filaments align themselves and other divisome components with the short axis of the cell. This process might be aided by FtsZ treadmilling, which could provide a long-range distribution mechanism^7,8,42^ (Figure 6a, middle).

We propose that curvature-sensing FtsA double filaments provide a solution to the FtsZ alignment problem of how FtsZ filaments align with the short cell axis during division^43^. Consistently, the fraction of directionally treadmilling FtsZ filaments decreases in a Δ*ftsA* strain of *B. subtilis*^10^. We hypothesise that FtsA double filaments and treadmilling FtsZ filaments align and evenly distribute divisome components in the narrow division plane. Most importantly, this could restrict movement of FtsWI, which is the bipartite PG synthase of the divisome, in such a way that cell-constricting septal PG synthesis follows the cell’s circumference (Figure 6a, right). FtsN might function as an activation switch of the divisome by coordinating activities of FtsA and FtsWI. Recently, a more direct interaction between FtsA and FtsW has also been proposed^44^.

We found that the short cytoplasmic tail of FtsN promotes FtsA double filament formation (Figure 2a, d and e), which adds to previous evidence reporting that FtsA can form different polymers^30,32,37^. Importantly, our structural data reveal that the FtsA double filament is compatible with FtsN binding. Double filament formation also positions FtsA’s IC domain close to the inner membrane (Extended Data Figure 1c), which might facilitate binding of divisome components such as FtsQ^24^.

Taken together with recent reports establishing FtsWI^45^ and RodA-PBP2^46,47^ as bipartite PG synthases, our data showing the similarities between FtsA and MreB double filaments strengthen the previously proposed evolutionary relationships between the divisome and elongasome^2^ (Figure 6b). In *Chlamydia*, one of the few bacteria that lack FtsZ, MreB has been implicated in the organisation of cell division^48^, which further suggests that divisome and elongasome share some basic functions.

Which of FtsZ’s many functions makes it the early organiser of the divisome, but are not required in the elongasome? Our model suggests that FtsZ is the long-range organiser of the division site, which ensures that septal PG synthases only function in a single division plane, and are evenly distributed around the cell’s circumference. FtsA aligns the PG synthases with the orientation of the division ring, so that septal PG glycan synthesis mediated by the divisome goes around the ring’s circumference.

Future studies will need to pinpoint when during cell division FtsA double filaments form. The difference in interaction partners between monomeric and polymeric FtsA, and how they influence each other, will need to be investigated concomitantly. These studies will deepen our understanding of the central role of FtsA polymerisation in FtsZ-based cell division and bring us closer to *in vitro* reconstitution of bacterial cell division.

## Online Methods

### Expression plasmids

Expression plasmids (Supplementary Table T3) were cloned using NEBuilder HiFi DNA Assembly Mix (NEB). *E. coli* MAX Efficiency DH5α (ThermoFisher) or C41(DE3) cells (Lucigen or Sigma) were used for plasmid propagation and protein expression, respectively. Plasmid sequences are provided in Supplementary Data D1.

### Protein expression and purification

The sequences of all proteins used in this study are listed in Supplementary Table T4. Purifications were carried out at 4-6°C unless stated otherwise. Buffers were prepared in Millipore water (MPW), pH-adjusted at room temperature (RT) and filtered through a 0.22 μm PES filter.

#### GST-SENP1

GST-SENP1 was expressed in C41(DE3) cells grown in 2xTY medium, supplemented with 100 μg/ml ampicillin, at 37°C. Cells were induced with 0.5 mM IPTG at OD_600_ = 0.6-0.8, grown over night at 18°C and harvested by centrifugation. Cells were lysed in buffer SA (50 mM Tris/HCl, 150 mM NaCl, 2 mM TCEP, 1 mM EDTA, 5 % glycerol, pH 8.5), supplemented with DNase, RNase and cOmplete EDTA-free Protease Inhibitor Cocktail (Roche), using a cell disruptor at 25 kpsi (Constant Systems). The lysate was centrifuged at 100,000x g for 30 min at 4°C. The supernatant was added to Glutathione Sepharose 4B beads (Cytiva) and incubated for 2h at 4°C. The beads were thoroughly washed in buffer SA, followed by buffer SA with 500 mM NaCl and, again, buffer SA. The protein was eluted in buffer SA with 10 mM reduced glutathione. Peak fractions were concentrated with a Vivaspin 20 concentrator (30 kDa MWCO, Sartorius). The protein was further purified by size exclusion chromatography on a HiLoad 26/600 Superdex 200 pg column (Cytiva), equilibrated in buffer SEC-S (50 mM Tris/HCl, 50 mM NaCl, 5 mM TCEP, 1 mM EDTA, 1 mM NaN_3_, 5 % glycerol, pH 8.0). Peak fractions were concentrated to ~15-20 mg/ml, frozen in aliquots and stored at −80°C.

#### 6H-TEV protease

6H-TEV protease was expressed in C41(DE3) cells grown in 2xTY medium, supplemented with 30 μg/ml kanamycin, at 37°C. Cells were induced with 1 mM IPTG at OD_600_ = 0.6-0.8, grown over night at 19°C and harvested by centrifugation. Cells were lysed in TEV lysis buffer (20 mM Tris/HCl, 500 mM NaCl, 1 mM TCEP, 1 mM NaN_3_, pH 8.0), supplemented with DNase and RNase, using a cell disruptor at 25 kpsi (Constant Systems). The lysate was centrifuged at 100,000x g for 30 min at 4°C. The supernatant was supplemented with 20 mM imidazole and loaded onto a HisTrap FF column (Cytiva). The column was washed with TEV lysis buffer supplemented with 20 mM imidazole. The protein was eluted in TEV lysis buffer with increasing concentrations of imidazole, concentrated with Vivaspin 20 concentrators (10 kDa MWCO, Sartorius) and further purified by size exclusion chromatography on a HiLoad 16/600 Superdex 75 pg column (Cytiva), equilibrated in TEV lysis buffer. Peak fractions were concentrated to 30 mg/ml, frozen in aliquots and stored at −80°C.

#### Full-length FtsAs

C-terminal intein-CBD-12H fusions of EcFtsA, EcFtsA^M96E, R153D^, EcFtsA^E124A^, EcFtsA^I143L^, EcFtsA^G50E^, EcFtsA^R286W^ or VmFtsA were expressed in C41(DE3) cells grown in 2xTY medium, supplemented with 100 μg/ml ampicillin, at 37°C. Cells were induced with 0.5 mM IPTG at OD_600_ = 0.8-1.0, grown over night at 18°C and harvested by centrifugation. Cells were lysed in buffer LB2 (50 mM Tris/HCl, 500 mM NaCl, 5 mM TCEP, 10 mM MgCl2, 1 mM NaN_3_, pH 8.0), supplemented with DNase, RNase and cOmplete EDTA-free Protease Inhibitor Cocktail (Roche), using a cell disruptor at 25 kpsi (Constant Systems). The lysate was centrifuged at 100,000x g for 30 min at 4°C. The supernatant was supplemented with 50 mM imidazole and loaded onto a HisTrap HP column (Cytiva). The column was washed with buffer SEC2 (50 mM CHES/KOH, 500 mM KCl, 5 mM TCEP, 10 mM MgCl2, 5 % glycerol, 1 mM NaN_3_, pH 9.0) supplemented with 100 mM imidazole. The protein was eluted in buffer SEC2 with increasing concentrations of imidazole and loaded onto chitin resin (NEB) packed in a XK 50/20 column (Cytiva). The column was thoroughly washed with buffer CHIT2 (buffer SEC2 with 1 mM EGTA), followed by buffer CHIT2 with 50 mM 2-mercaptoethanol (2-ME) and incubated over night at 4°C without flow, allowing for intein cleavage. Cleaved FtsA was eluted in buffer CHIT2 with 50 mM 2-ME, followed by buffer CHIT2, concentrated with Vivaspin 20 concentrators (30 kDa MWCO, Sartorius) and further purified by size exclusion chromatography on a HiLoad 16/600 Superdex 200 pg column (Cytiva), equilibrated in buffer SEC2. Peak fractions were concentrated to 7-8 mg/ml, frozen in aliquots and stored at −80°C. Protein mass was verified by ESI-TOF mass spectrometry.

#### C-terminally truncated FtsAs

N-terminal 6H-SUMO fusions of EcFtsA^1-405^, XpFtsA^1-396^ or VmFtsA^1-396^ were expressed in C41(DE3) cells grown in 2xTY medium, supplemented with 30 μg/ml kanamycin, at 37°C. Cells were induced with 0.5 mM IPTG at OD_600_ = 0.8-1.0, grown for 5h at 37°C (6H-SUMO-XpFtsA^1-396^) or over night at 25°C (6H-SUMO-EcFtsA^1-405^ and 6H-SUMO-VmFtsA^1-396^) and harvested by centrifugation. Cells were lysed in buffer LB2, supplemented with DNase, RNase and cOmplete EDTA-free Protease Inhibitor Cocktail (Roche), using a cell disruptor at 25 kpsi (Constant Systems). The lysate was centrifuged at 100,000x g for 30 min at 4°C. The supernatant was supplemented with 20 mM imidazole and loaded onto a HisTrap HP column (Cytiva). The column was washed with buffer SEC2 with 20 mM imidazole. The protein was eluted in buffer SEC2 with increasing concentrations of imidazole, mixed with Glutathione Sepharose 4B beads (Cytiva) and purified GST-SENP1, and incubated over night at 4°C. The sample was passed through a gravity flow column and concentrated using Vivaspin 20 concentrators (30 kDa MWCO, Sartorius). Concentrated protein was further purified by size exclusion chromatography on a HiLoad 26/600 Superdex 200 pg column (Cytiva), equilibrated in buffer SEC2 for EcFtsA^1-405^ or crystallisation buffer (20 mM CHES/KOH, 100 mM KCl, 5 mM TCEP, 5 mM MgCl_2_, 5 % glycerol, 1 mM NaN_3_, pH 9.0) for XpFtsA^1-396^ and VmFtsA^1-396^. Peak fractions were concentrated to 12-19 mg/ml, frozen in aliquots and stored at −80°C. Protein mass was verified by ESI-TOF mass spectrometry.

#### Isotope-labelled VmFtsN^1-29^-ENLYFQ and Gly-Gly-VmFtsN^2-29^

Isotope-labelled VmFtsN^1-29^-TEV-lipoyl tag-6H (resulting in VmFtsN^1-29^-ENLYFQ) and 6H-lipoyl tag-TEV-G-VmFtsN^2-29^ (resulting in GG-VmFtsN^2-29^) were expressed in C41(DE3) cells in M9 medium (6 g/L Na_2_HPO_4_, 3 g/L KH_2_PO_4_, 0.5 g/L NaCl) supplemented with 100 μg/ml ampicillin and 1.7 g/L yeast nitrogen base without NH_4_Cl and amino acids (Sigma Y1251) at 37°C. 1 g/L ^15^NH_4_Cl and 4 g/L ^13^C-glucose were supplemented for ^15^N and ^13^C labelling, respectively. Cells were induced with 0.5 mM IPTG at OD_600_ = 0.6-0.8., grown over night at 25°C and harvested by centrifugation. Cells were lysed in buffer LB5 (50 mM HEPES/KOH, 250 mM KCl, 1 mM TCEP, pH 7.7), supplemented with DNase, RNase and cOmplete EDTA-free Protease Inhibitor Cocktail (Roche), using a cell disruptor at 25 kpsi (Constant Systems). The lysate was centrifuged at 100,000x g for 30 min at 4°C. 20 mM imidazole was added to the supernatant before loading onto a HisTrap HP column (Cytiva). The column was washed with buffer LB5 with 20 mM or 50 mM imidazole for VmFtsN^1-29^-TEV-lipoyl tag-6H and 6H-lipoyl tag-TEV-G-VmFtsN^2-29^, respectively. Proteins were eluted in buffer LB5 with increasing concentrations of imidazole, mixed with 6H-TEV protease and incubated over night at 4°C. Samples were diluted in buffer LB5 to a final imidazole concentration of ~50 mM and loaded onto a HisTrap HP column equilibrated in buffer LB5 with 50 mM imidazole. The column was washed with buffer LB5 supplemented with 50 mM imidazole. Flow-through and wash fractions were concentrated using Vivaspin 15R concentrators (2 kDa MWCO HY, Sartorius). For Gly-Gly-VmFtsN^2-29^ only, the sample was further diluted in buffer SA2 (50 mM HEPES/KOH, 50 mM KCl, 1 mM TCEP, pH 7.7) to a final salt concentration of ≤ 100 mM and loaded onto a HiTrap SP HP column (Cytiva). The sample was eluted in a linear gradient of buffer SA2 with 1000 M KCl and afterwards concentrated.

Concentrated VmFtsN^1-29^-ENLYFQ or Gly-Gly-VmFtsN^2-29^ was further purified by size exclusion chromatography on a Superdex Peptide 10/300 GL column (Cytiva), equilibrated in NMR buffer (50 mM MES/KOH, 50 mM KCl, 1 mM TCEP, pH 6.0). Peak fractions were concentrated to 2-3 mg/ml, frozen in aliquots and stored at −80°C.

Protein mass was verified by ESI-TOF mass spectrometry.

#### FtsN peptides

FtsN peptides were chemically synthesised by Generon/Neobiotech. Purity (≥ 95 % in HPLC) and molecular mass were verified by the company. Lyophilised peptides were resuspended in binding buffer (50 mM HEPES/KOH, 100 mM KAc, 5 mM MgAc_2_, pH 7.7). Stock concentrations were determined using an ND-1000 spectrophotometer (NanoDrop Technologies) or a Direct Detect Infrared Spectrometer (Merck Millipore) for peptides without tyrosine and tryptophan residues. Stock concentrations were in the range of 20-70 mM. Peptide sequences are given in Supplementary Table T5.

### Crystal structure determination

Crystallisation conditions were screened using our in-house high-throughput crystallisation facility^49^. EcFtsA^1-405^ at 7 mg/ml, XpFtsA^1-396^ at 7 mg/ml or VmFtsA^1-396^ at 5 mg/ml was mixed with 2 mM ATP. For co-crystallisation, VmFtsA^1-396^ at 3 mg/ml was mixed with 2 mM ATP and 0.353 mM VmFtsN^1-29^. For EcFtsA^1-405^ and XpFtsA^1-396^, 100 nl protein solution and 100 nl of the crystallisation solutions were mixed in MRC sitting drop crystallisation plates for vapour diffusion. 500 nl protein solution and 500 nl of crystallisation solutions were used to obtain optimised co-crystals of VmFtsA^1-396^ with VmFtsN^1-29^. Optimisation for VmFtsA^1-396^ followed the idea of an “anticipated optimisation approach”^50^. 250 nl of protein solution were mixed with 200 nl of initial crystallisation solution (31.55 % v/v PEG 400, 0.21 M MgCl_2_, 0.1 M Tris/HCl pH 8.5) and 50 nl of follow-up crystallisation solution (10 % v/v 2-propanol, 0.2 M CaAc_2_, 0.1 M MES/NaOH pH 6.0). Plates were incubated at 21°C. 1-2 μl of cryoprotectant solution were added to crystals shortly before mounting single crystals and flash-freezing them in liquid nitrogen. VmFtsA^1-396^-FtsN^1-29^ co-crystals were soaked in cryoprotectant solution for 1h prior to mounting. Optimised conditions yielding crystals and cryoprotectant solutions are listed in Supplementary Table T1. Diffraction data were collected on single crystals at Diamond Light Source (DLS), Harwell, UK, at 100 K using the in-house Generic Data Acquisition (GDA) software, as indicated in Supplementary Table T1. Diffraction data were processed using the CCP4 suite^51^. Initial phases were obtained by molecular replacement using PHASER 2.82 and 2.83^52^. Search models are listed in Supplementary Table T1. Models were rebuilt using MAIN 2017^53^ and COOT 0.8.9.3^54^ and refined using REFMAC 5.8^55^ and PHENIX 1.17.1^56^. Final statistics are summarised in Supplementary Table T1, and the structure factors as well as atomic coordinates have been deposited in the Protein Data Bank (PDB) with accession codes 7Q6D, 7Q6G, 7Q6F and 7Q6I. Note that VmFtsN^1-29^ (chains X and Y) density was modelled as VmFtsN^1-8^ for refinement of PDB 7Q6I but deposited as unknown poly-Ala. Structure factors and atomic coordinates for our interpretation of the VmFtsN^1-29^ density (Extended Data Figure 3d) are provided in Supplementary Data D2.

### SPR

SPR was performed using a Biacore T200 using CM5-sensor chips (Cytiva). Reference control and analyte channels were equilibrated in binding buffer. FtsA was immobilised onto the chip surface via amide coupling using the supplied kit (Cytiva) to reach a RU value of between 2000 and 7800 RU for separate experiments. Analytes were injected for 120 s followed by a 300 s dissociation in a 1:2 dilution series with initial concentrations of: 10 μM for peptide EcFtsN^1-32^ (Figure 2b); 60 μM for VmFtsN^1-29^ (Extended Data Figure 2b); 20 μM for FtsN peptides in Extended Data Figure 2i. After reference and buffer signal correction, sensogram data were fitted using Prism 8.0 (GraphPad). Equilibrium response (*R_eq_*) data were fitted to a single-site interaction model to determine *K_d_*:

(1) $$R_{eq}=\left(\frac{CR_{\max}}{C+K_{d}}\right)+B,$$

where *C* is the analyte concentration and *R_max_* is the maximum response at saturation and *B* is the background resonance.

### FP

Peptides EcFtsN^1-32^-C and VmFtsN^1-29^-C were labelled with maleimide-Atto 495 (Merck Life Science UK). EcFtsA^1-405^ and VmFtsA^1-396^ were buffer exchanged into binding buffer using Zeba Spin Desalting Columns (7 kDa MWCO, ThermoFisher) prior to FP experiments. 20 nM labelled peptide was mixed 1:1 with a 1:2 dilution series of protein with initial concentrations of 40 μM for EcFtsA^1-405^ or 120 μM for VmFtsA^1-396^ in binding buffer supplemented with 0.05 % v/v Tween-20 in a 384-well low flange black flat bottom non-binding surface microplate (Corning). Reactions were prepared in triplicate. Fluorescence polarisation was measured using a PHERAstar FSX (BMG Labtech) directly after reaction setup and after incubation at RT for 30 min and 2 h to ensure equilibrium had been reached. Data were fitted using Prism 8.0 (GraphPad). Dissociation constants were calculated using a two-step model:

(3) $$F=F_{0}+\frac{F_{LO}\cdot [P_{T}]}{\left[K_{DLO}]+[P_{T}\right]}+\frac{F_{Hi}\cdot [P_{T}]}{\left[K_{DHi}]+[P_{T}\right]},$$

where *F*_0_ is the anisotropy in the absence of titrating protein, [*P*_T_] is the total concentration of protein, and *F*_Lo_ and *F*_Hi_ are the anisotropy changes at saturation of low and high affinity sites with binding constants of *K_DLo_* and *K_DHi_*, respectively.

### FDS-AUC

Peptides EcFtsN^1-32^-C and VmFtsN^1-29^-C were labelled with maleimide-Atto 495 (Merck Life Science UK). EcFtsA^1-405^ and VmFtsA^1-396^ were buffer exchanged into binding buffer using Zeba Spin Desalting Columns (7 kDa MWCO, ThermoFisher) before experiments. 20 nM labelled peptide was mixed 1:1 with a 1:3 dilution series of protein with initial concentrations of 90 μM for EcFtsA^1-405^ or 120 μM for VmFtsA^1-396^ in binding buffer supplemented with 0.05 % v/v Tween-20. Samples were centrifuged at 163,000x g at 20°C in an An50Ti rotor using an Optima XL-I analytical ultracentrifuge (Beckman) equipped with a fluorescence optical system (Aviv Biomedical) with fixed excitation at 488 nm and fluorescence detection at > 505 nm. Data were processed and analysed using SEDFIT 16 and SEDPHAT 15.2^57^ following the protocol for high-affinity interactions detected by fluorescence^58^. Binding constants were estimated using a two-step model. Data were plotted using GUSSI 1.4.2^59^.

### Co-pelleting assay

EcFtsA^1-405^ or VmFtsA^1-396^ at 1 mg/ml (~23 μM) alone or mixed with half, 1-, 3-, 6- or 10-fold the molar concentration of EcFtsN^1-32^ or VmFtsN^1-29^, respectively, in binding buffer was incubated at 20°C for 5-15 min before centrifugation at 20,000x g for 10 min. Supernatant and pellet were separated carefully and analysed by SDS-PAGE. Protein band intensities were quantified using ImageJ 2.1.0^60^.

### Lipid monolayers and negative stain electron microscopy

Lipid monolayers were prepared from *E. coli* polar lipid extract (Avanti)^61^. Wells of a custom-made Teflon block were filled with 60 μl binding buffer. 20 μg of lipids, dissolved in chloroform, were applied on top of the buffer, and incubated for 2 min. Next, baked (60°C, overnight) CF300-Cu-UL EM grids (EMS) were placed on top of the wells with the carbon side facing downwards. Grids were incubated for 20-60 min. FtsA at 0.2 mg/ml for EcFtsA^M96E,^ ^R153D^ and VmFtsA or at 0.1 mg/ml for all other FtsA variants was mixed with 1 mM ATP and indicated FtsN peptides at 10-fold molar excess, unless stated otherwise, in binding buffer. Samples were incubated for 10 min at RT. EM grids were carefully lifted off the buffer and blotted from the side. 4 μl sample were applied to the grid and incubated for 30 s before staining with 2% w/v uranyl formate. Grids were imaged on a Tecnai Spirit electron microscope (ThermoFisher) operating at 120 kV and equipped with a Gatan Orius SC200W camera. Presented micrographs were contrast adjusted and blurred for display purposes.

### Cryo-EM of EcFtsA “mini-rings” and EcFtsA-FtsN^1-32^ double filaments on lipid monolayer

Lipid monolayers were prepared as described above, with the exception that Quantifoil Au R0.6/1 300 mesh grids (Quantifoil) were used. For “mini-rings” (Extended Data Figure 3b), EcFtsA at 0.15 mg/ml was mixed with 1 mM ATP in binding buffer, and incubated for 30 min at RT. For double filaments (Figure 2f), EcFtsA at 0.1 mg/ml was mixed with 1 mM ATP and EcFtsN^1-32^ at 22 μM in binding buffer, and incubated for 20 min at RT. Grids were gently blotted from the side after attachment of the monolayer and inserted into a Vitrobot Mark III (ThermoFisher) set to 20°C and 100% humidity. 3 μl of sample were applied to the grid, incubated for 30 s and blotted for 12-15 s (0.5 s drain time, -15 blot force) before plunge-freezing into liquid ethane maintained at −180°C using a cryostat^62^. For “mini-rings”, grids were imaged using a Titan Krios (ThermoFisher) operating at 300 kV and equipped with a Quantum energy filter (Gatan) set to 20 eV slit width. Movies were collected on a K2-XP direct electron detector at a pixel size of 2.32 Å, −3 to −5 μm defocus and a total dose of 25 e^−^/Å^2^ using EPU 2.13 (ThermoFisher). For double filaments, grids were imaged using a Tecnai G2 Polara (ThermoFisher) operating at 300 kV. Movies were collected on a Falcon III direct electron detector at a pixel size of 1.38 Å, −3.3 to −4 μm defocus and a total dose of 100 e^−^/Å^2^ using EPU 1.5 (ThermoFisher). Data were processed using MotionCor2^63^, CTFFIND4^64^ and RELION-3.0^65^ for double filaments or RELION-4.0^66^ for “mini-rings”. A total of 162,725 and 104,660 particles were automatically picked and extracted for “mini-rings” and double filaments, respectively, with the presented 2D class averages corresponding to 14,067 and 14,602 particles, respectively. Presented images were upscaled (Extended Data Figure 3b), contrast adjusted and blurred for display purposes.

### HDX-MS

VmFtsA^1-396^ at 10 μM was mixed with 2 mM ATP and, if indicated, VmFtsN^1-29^ at 3- or 10-fold molar excess in binding buffer. 5 μL of sample were added to 40 μL of D_2_O buffer at RT for 3, 30, 300 and 1800 seconds, then quenched and frozen until further processing. Samples were rapidly thawed and subjected to pepsin cleavage followed by reversed phase HPLC separation. The protein was passed through a 2.1 x 30 mm, 5 μm Enzymate BEH immobilised pepsin column (Waters) at 200 μL/min for 2 min. Peptic peptides were trapped and desalted on a 2.1 x 5 mm C18 trap column (Acquity BEH C18 Van-guard pre-column, 1.7 μm, Waters). Peptides were eluted over 12 min using a 5-36% gradient of acetonitrile in 0.1% v/v formic acid at 40 μL/min. Peptides were separated on a 100 mm x 1 mm, 1.7 μm Acquity UPLC BEH C18 reverse phase column (Waters). Peptides were detected on a SYNAPTG2-Si HDMS mass spectrometer (Waters) acquiring over a *m/z* range of 300 to 2000, with standard electrospray ionisation (ESI) source and lock mass calibrated using [Glu1]-fibrino peptide B (50 fmol/μL). The mass spectrometer was operated at a source temperature of 80°C and a spray voltage of 2.6 kV. Spectra were collected in positive ion mode. Peptides were identified by MS^e^^67^ using a 5-36% gradient of acetonitrile in 0.1% v/v formic acid over 12 min. The resulting MS data were analysed using Protein Lynx Global Server 3.0.3 (Waters) with an MS tolerance of 5 ppm. Mass analysis of the peptide centroids was performed using DynamX 3.0 (Waters). Only peptides with a score > 6.4 were considered. The first round of analysis and identification was performed automatically using DynamX 3.0; however, all peptides (deuterated and non-deuterated) were manually verified at every time point for the correct charge state, presence of overlapping peptides, and correct retention time. Deuterium incorporation was not corrected for back-exchange and represents relative, rather than absolute changes in deuterium levels. Changes in H/D amide exchange in any peptide may be due to a single or multiple amides within that peptide. Time points were prepared in parallel and data for individual time points were acquired on the mass spectrometer on the same day.

### NMR

Backbone amide peaks of VmFtsN^1-29^-ENLYFQ were assigned using 167 μM ^15^N, ^13^C-labelled peptide at 278 K in NMR buffer. Standard triple resonance spectra: HNCO, HN(CA)CO, HNCACB, and CBCA(CO)NH (Bruker) were collected with 20 % non-uniform sampling and processed with compressed sensing using MddNMR 3.2^68^. Backbone resonances were assigned using MARS 1.2^69^. Topspin 3.6.0 (Bruker) was used for processing of 2D data and NMRFAM-Sparky 1.47^70^ was used for spectra analysis. Assignment of VmFtsN^1-29^-ENLYFQ was transferred and extended to Gly-Gly-VmFtsN^2-29^ using 295 μM ^15^N-labelled peptide at 278 K in NMR buffer. The first glycine (G0) of Gly-Gly-VmFtsN^2-29^ was not observed.

For binding studies, ^1^H, ^15^N BEST-TROSY spectra were acquired at 278 K on 50 μM Gly-Gly-VmFtsN^2-29^ mixed with equimolar concentration of VmFtsA^1-396^ in NMR buffer. As sensitivity was compromised by the formation of FtsA polymers upon binding of FtsN peptide, multiple short experiments were acquired and added up to define the ideal time window for data analysis. Each spectrum was acquired with 128 scans and a recycle delay of 400 ms, with a final spectral resolution of 4.7 Hz per points. Relative peak intensities were normalised to the C-terminal residue R29 of Gly-Gly-VmFtsN^2-29^ and analysed as I_bound_/I_free_, with I_bound_ and I_free_ being the peak intensities of Gly-Gly-VmFtsN^2-29^ with (bound) and without (free) VmFtsA^1-396^, respectively.

### EcFtsA “mini-rings” and EcFtsA-FtsN^1-32^ filaments on liposomes

Liposomes were prepared from *E. coli* polar lipid extract (Avanti) by extrusion using a Mini Extruder fitted with a polycarbonate membrane with 0.4 μm (Figure 4b) or 1 μm (Figure 4a, c) pore size (Avanti) in binding buffer (Figure 4b) or binding buffer without magnesium for EcFtsA “mini-rings” and EcFtsA-EcFtsN^1-32^ filaments (Figure 4a, c), respectively. Preformed liposomes at 1 mg/ml (Figure 4a, c) or 2 mg/ml (Figure 4b) were mixed with 0.5 mM MgATP (Figure 4a, c) or 1 mM MgATP (Figure 4b) and proteins at the following concentrations: no proteins (Figure 4a), FtsA at 20 μM (Figure 4b), FtsA at 20 μM and FtsN^1-32^ at 200 μM (Figure 4c, left) and FtsA at 5 μM and FtsN^1-32^ at 50 μM (Figure 4c, right). Samples were incubated at RT for 30 min without proteins or 10 min with proteins, respectively. 3 μl of sample were applied to a freshly glow-discharged Quantifoil Cu/Rh R2/2 200 mesh grid (Quantifoil) (Figure 4b) or Quantifoil Au R2/2 200 mesh grid (Quantifoil) (Figure 4a, c), blotted for 3.5-7.5 s (0.5 s drain time, -15 blot force) and plunge-frozen into liquid ethane maintained at −180°C using a cryostat^62^ using a Vitrobot Mark III (ThermoFisher) set to 20°C and 100% humidity. Grids were imaged on a Tecnai F20 (ThermoFisher) equipped with a Falcon II direct electron detector or a Glacios (ThermoFisher) equipped with a Falcon III detector. Microscopes were operated at 200 kV and cryogenic temperature. Presented micrographs were motion-corrected (if collected on Glacios), contrast adjusted and blurred for display purposes.

### EcFtsA-FtsN^1-32^ filaments inside liposomes

EcFtsA-FtsN^1-32^ filaments were encapsulated into liposomes by dilution of CHAPS-solubilised *E. coli* total lipid extract (Avanti)^40^. 50 μl of preincubated FtsA at 20 μM mixed with 200 μM FtsN^1-32^ and 0.5 mM MgATP in binding buffer without magnesium were added to 50 μl of *E. coli* total lipid extract (Avanti) solubilised at 10 mg/ml in binding buffer without magnesium supplemented with 20 mM CHAPS. The sample was incubated at RT for 35 min, before it was gradually diluted with 500 μl of binding buffer without magnesium supplemented with 0.5 mM MgATP within 20 min. 3 μl of sample were applied to a freshly glow-discharged Quantifoil Au R2/2 200 mesh grid (Quantifoil), blotted for 5.5-7.5 s (0.5 s drain time, -15 blot force) and plunge-frozen into liquid ethane maintained at −180°C using a cryostat^62^ using a Vitrobot Mark III (ThermoFisher) set to 20°C and 100% humidity. Grids were imaged on a Tecnai F20 (ThermoFisher) equipped with a Falcon II direct electron detector, operating at 200 kV and cryogenic temperature. Rarely, EcFtsA-FtsN^1-32^ filaments were observed inside liposomes when added to the outside of preformed liposomes (Figure 4f), most likely due to membrane rearrangements during handling. Here, preformed liposomes extruded to 1 μm at 1 mg/ml were mixed with 0.5 mM MgATP, FtsA at 2.5 μM and FtsN^1-32^ at 25 μM. Presented micrographs were contrast adjusted and blurred for display purposes. For 2D class averages (Figure 4g), movies were collected on a Glacios with a Falcon III direct electron detector at a pixel size of 1.99 Å, −2.5 to −4 μm defocus and a total dose of 56 e ^−^/Å^2^ using SerialEM 3.9^71^. Data were processed using MotionCor2^63^, CTFFIND4.1^64^ and RELION-3.1^65^. Presented images were upscaled and blurred for display purposes.

### Strain construction

A cloning and recombination strategy based on REXER^72^ was used as illustrated in Extended Data Figure 4a. The helper plasmid pKW20 (NCBI ID: MN927219.1)^72^ was used for genome engineering. Acceptor strain SFB123 was created by integrating a *pheS^T251A,A294G^-hygR* double selection cassette downstream of the *lpxC* gene using *λ*-Red recombineering^73^. pheS^T251A,A294G^/pheS* confers toxicity through misincorporation of 4-chloro-phenylalanine (4-CP) during translation^74^. We found a long (5 kB) homologous region upstream of FtsA to benefit recombination efficiency. SFB143, an *E. coli* MDS42 *thi^-^* strain transformed with the non-transferrable conjugative plasmid pJF146 (NCBI ID: MK809154.1)^75^, was used as donor strain during conjugation. SFB143 cells were made chemically competent^76^.

Shuttle plasmid pFB483 was designed with a pMB1 origin of replication, a *pheS^T251A,A294G^-hygR* double selection cassette, a CRISPR array targeting *ftsW* and the region upstream of *secM*, and a *ccdB* toxin gene (outsert) flanked by *Bsa*I acceptor sites for Golden Gate assembly^77^. CRISPR arrays were designed to mediate scarless excision. pFB483 was propagated in a *ccdB* survival strain.

Targeting constructs were split into 2-3 modules for insertion of single or double point mutations, respectively. Initially, the internal 3x HA-tag (120 bp including a *Xho*I restriction site) was inserted into *ftsA* using three modules, resulting in sTN001. Point mutations were introduced by PCR using sTN001 as template, and modules were assembled into pFB483 via Golden Gate assembly with *Bsa*I. The final targeting construct also introduced a kanamycin-selectable *neoR* marker downstream of the *lpxC* gene. Assembled shuttle vectors were transformed into SFB143 and selected on LB agar plates supplemented with 200 μg/ml hygromycin-B and 50 μg/ml apramycin at 37°C.

Acceptor cells (SFB123) were grown to stationary phase in 5 ml LB supplemented with 10 μg/ml tetracycline. 4 ml of culture were harvested by centrifugation and washed three times in LB, before being transferred into 50 ml LB supplemented with 10 μg/ml tetracycline and 0.5 % w/v L-arabinose. Cells were grown at 37°C for 1h, harvested and washed three times in LB. In the meantime, donor transformants were washed off the agar plates using 2 ml LB and left at RT. All cultures were resuspended in LB to an OD_600_ of 40. 12.5 μl of acceptor cells were mixed 87.5 μl of donor cells and spotted onto well-dried TYE plates. Spots were air-dried, before plates were incubated at 30°C for 1h. Cells were washed off the plates with LB and transferred into 50 ml LB supplemented with 12.5 μg/ml kanamycin and 10 μg/ml tetracycline. Cells were grown at 37°C for 4h, harvested and plated on LB agar plates supplemented with 12.5 μg/ml kanamycin, 10 μg/ml tetracycline, 2 % glucose and 2.5 mM 4-CP. Strains were single colony purified, and verified by marker analysis and colony PCR followed by *Xho*I digestion and Sanger sequencing. Strains with desired point mutations were cured of pKW20 by repeated growth in LB in absence of antibiotics, diluted 1:10^6^ and plated on TYE plates. Strains were verified by marker analysis and Sanger sequencing of PCR products covering the targeting region. Strains used in Figure 5c were further whole genome sequenced on a MiSeq (Illumina). NGS data were analysed using breseq v0.35.1^78^.

Strains are listed in Supplementary Table T6. Annotated shuttle vectors and genomic loci are provided in Supplementary Data D1 and D3, respectively.

### Assessment of growth and cell elongation phenotypes

#### Growth on solid media

Strains were streaked on the same TYE plate and incubated at 37°C overnight. The next morning, strains were re-streaked on a fresh TYE plate and incubated at 37°C for 12h.

#### Growth in liquid media

Strains were grown overnight in LB at 37°C. Cells were diluted 1/1000 into fresh LB into a 96-well flat bottom plate in octuplicate. The plate was incubated at 37°C in a Tecan microplate reader with regular shaking. Absorbance at 600 nm wavelength was measured every 5 min for 24h. OD_600_ values were background corrected, normalised to the maximum OD_600_ value of each well and averaged. Individual data points and the means were plotted.

#### DIC imaging of exponential phase cultures

Strains were grown overnight in LB at 37°C. The next day, cells were diluted 1/1000 into fresh LB and incubated at 37°C. 2-3 μl of exponential phase cells (OD_600_ = 0.2-0.3) were applied onto an agarose pad and imaged on a Nikon Eclipse E800 microscope equipped with a 100x oil objective and a Photometrics Iris 9 CMOS camera using a differential interference contrast (DIC) imaging setup. Presented images were contrast-adjusted for display purposes.

### In vivo cysteine cross-linking

Strains were grown to exponential phase (OD_600_ = 0.2-0.4) in LB from overnight cultures, and 0.9375 OD units harvested using centrifugation at 4°C. Cells were kept on ice for the duration of the experiment, unless stated otherwise. Cells were washed in 500 μl PBS and resuspended in 50 μl PBS. 1.25 μl DMSO or cross-linker (dBBr: Dibromobimane, BMOE: Bismaleimidoethane, BMB: 1,4-bismaleimidobutane or BMH: Bismaleimidohexane) in DMSO (20 mM stock) were added. Samples were incubated for 10 min and quenched by adding 1 μl 2-ME (1.43 M stock in MPW). Cells were resuspended in 50 μl lysis buffer [1 mM EDTA (pH 7.4), 14.3 mM 2-ME, cOmplete EDTA-free Protease Inhibitor Cocktail (Roche), 0.25 U/μl Benzonase (Merck) and 0.5 U/μl ReadyLyse Lysozyme (Lucigen) in B-PER (ThermoFisher)]. Samples were incubated at RT for 5 min. 50 μl of 2x SDS sample loading buffer supplemented with 3 % v/v 2-ME were added. Samples were incubated at 95°C for 5 min, and the equivalent of 0.1875 OD units of cells was analysed by SDS-PAGE. Western blotting was performed using a Trans-Blot turbo (BioRad) with the corresponding Midi 0.2 μm PVDF transfer pack. Blots were run at 25 V and 2.5 A for 7 min. Membranes were blocked in PBS + 5 % milk for 30-40 min, washed in PBS and incubated with anti-HA-Peroxidase antibody (Roche 12013819001) (1/1000 in PBST + 5 % milk) at RT for 1-1.5h. Membranes were washed thoroughly with PBST, developed using the ECL Prime Western blotting detection kit (Amersham/Cytiva) and imaged on a Gel DocTM XR+ (Bio-Rad). Presented images were contrast-adjusted for display purposes.

## Extended Data

## Supplementary Material

Source Data Extended Data Figure 2_gels

Source Data Extended Data Figure 2_numerical

Source Data Extended Data Figure 3

Source Data Extended Data Figure 4

Source Data Extended Data Figure 5

Source Data Figure 2_gel

Source Data Figure 2_numerical

Source Data Figure 5

Supplementary Data D1

Supplementary Data D2

Supplementary Data D3

Supplementary Data D4

Supplementary Information

Supplementary Movie M1

InventoryOfSupplementaryMaterial PMC Europe

## Data Availability

Atomic coordinates have been deposited in the PDB with accession codes 7Q6D (*Escherichia coli* FtsA^1-405^), 7Q6G (*Xenorhabdus poinarii* FtsA^1-396^), 7Q6F (*Vibrio maritimus* FtsA^1-396^, antiparallel double filament) and 7Q6I (*Vibrio maritimus* FtsA^1-396^ and FtsN^1-29^, bent tetramers in antiparallel double filament arrangement). NGS data associated with this study are available from the Sequence Read Archive at BioProject PRJNA852398. PDB entries 1E4F, 1E4G, 2YCH, 3WQT, 3WQU, 3WT0, 4A2A, 4A2B, 4CZJ were used for structural superpositions and analyses.
