# Supplementary figures and images for: Bacterial divisome protein FtsA forms curved antiparallel double filaments upon binding FtsN
Source: Nat Microbiol. Author manuscript; Available in PMC 2022 Dec 14. (PMC7613929; doi:10.1038/s41564-022-01206-9)

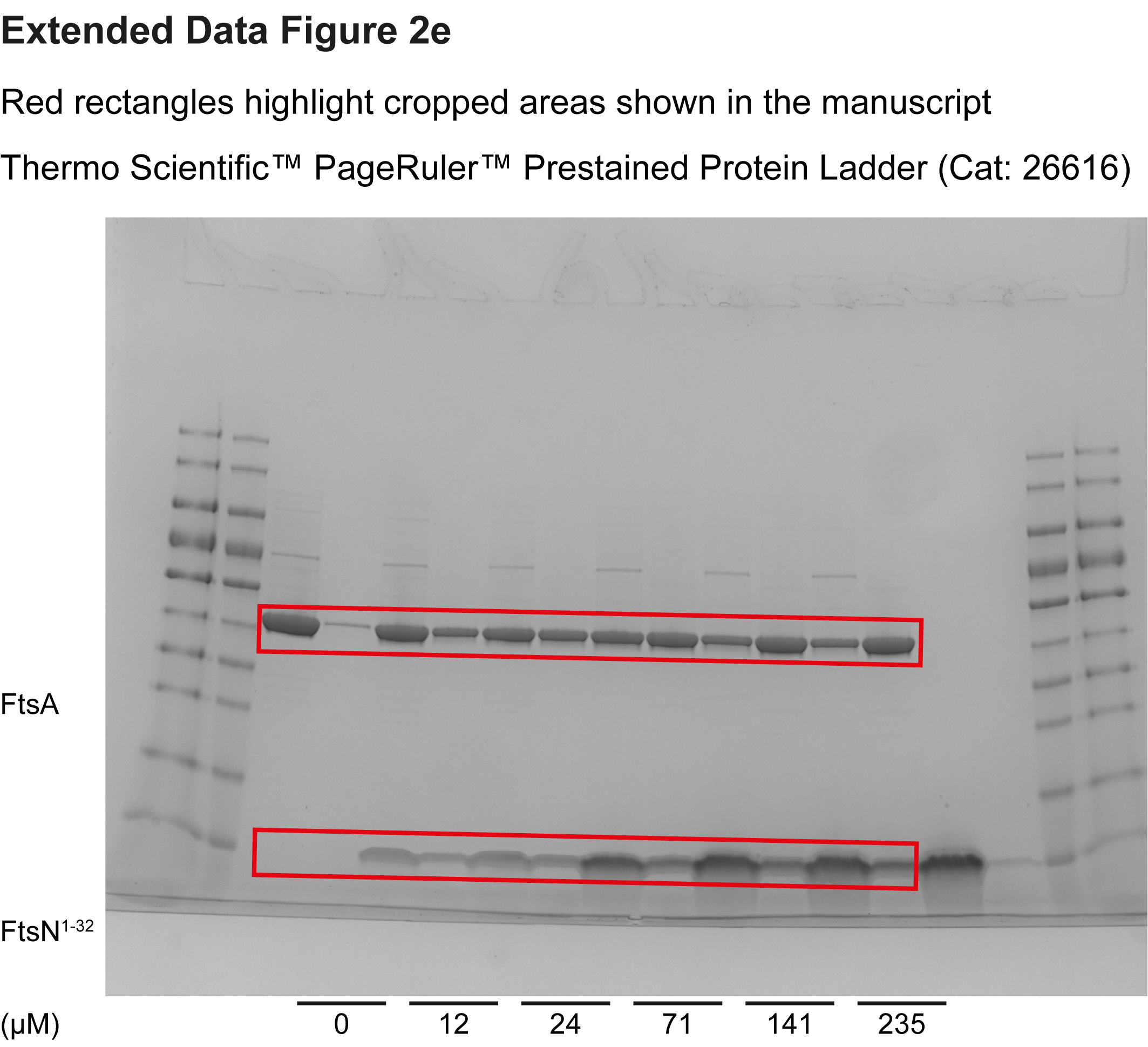

Supplement: Source Data Extended Data Figure 2_gels [file EMS151223-supplement-Source_Data_Extended_Data_Figure_2_gels.tif]

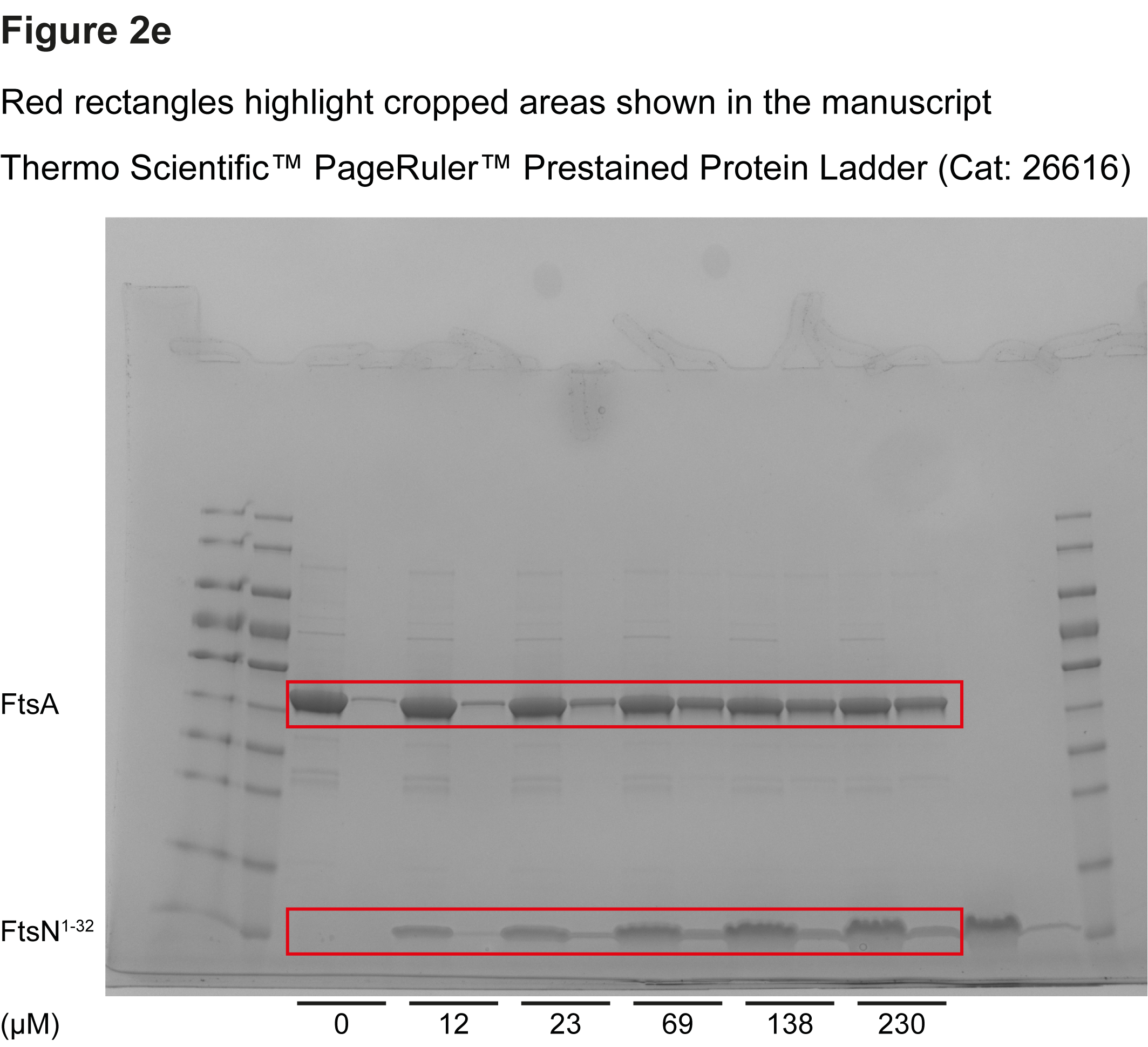

Supplement: Source Data Figure 2_gel [file EMS151223-supplement-Source_Data_Figure_2_gel.tif]

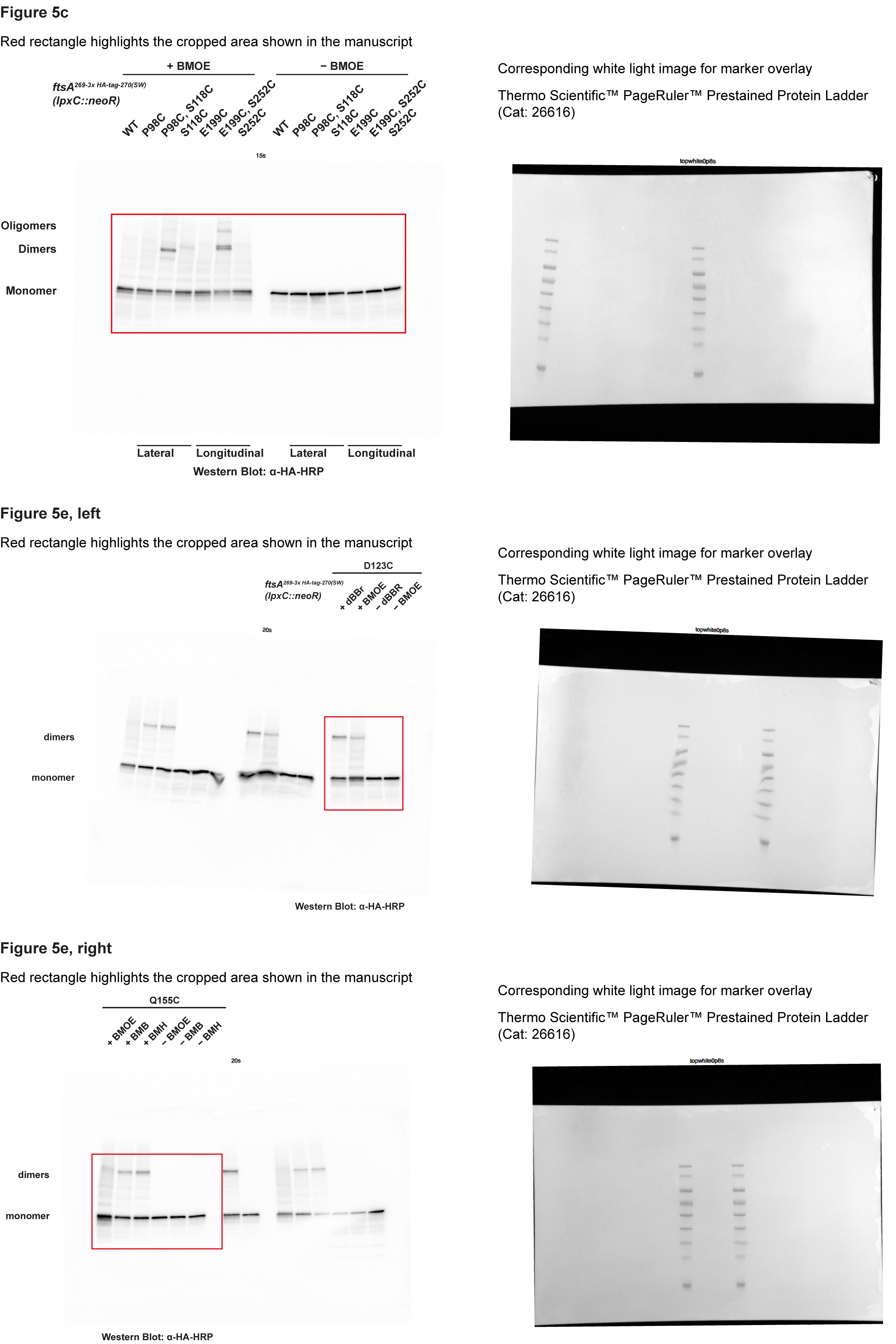

Supplement: Source Data Figure 5 [file EMS151223-supplement-Source_Data_Figure_5.tif]
