# Supplementary material for: Bacterial divisome protein FtsA forms curved antiparallel double filaments upon binding FtsN
Source: Nat Microbiol. Author manuscript; Available in PMC 2022 Dec 14. (PMC7613929; doi:10.1038/s41564-022-01206-9)
Supplement: Supplementary Information [file EMS151223-supplement-Supplementary_Information.pdf]

SUPPLEMENTARY INFORMATION

Bacterial divisome protein FtsA forms curved antiparallel double filaments upon binding FtsN

Tim Nierhaus[1], Stephen H McLaughlin[1], Frank Bürmann[1], Danguole Kureisaite-Ciziene[1], Sarah L Maslen[1,2], J Mark Skehel[1,2], Conny WH Yu[1], Stefan MV Freund[1], Louise FH Funke[1], Jason W Chin[1], Jan Löwe[1]*

[1] MRC Laboratory of Molecular Biology, Cambridge, UK.

[2] Present address: The Francis Crick Institute, London, UK.

*Corresponding author: Jan Löwe, MRC Laboratory of Molecular Biology, Cambridge Biomedical Campus, Francis Crick Avenue, Cambridge CB2 0QH, UK, email: jyl@mrc-lmb.cam.ac.uk, phone: +44 (0)1223 267064

CONTENTS

Supplementary Tables T1-6

Supplementary References

SUPPLEMENTARY TABLES

Supplementary Table T1. Crystallographic data.

**Statistics**				
**Sample**	***Escherichia coli FtsA***	***Xenorhabdus poinarii FtsA***	***Vibrio maritimus FtsA (double filament)***	***Vibrio maritimus FtsA and FtsN$^{1-29}$ (bent tetramers, some with FtsN$^{1-29}$ density)[4]***
NCBI database ID	AMK99051.1	WP_045957782.1	WP_042501243.1	FtsA: WP_042501243.1, FtsN: WP_042502685.1
Constructs	1-405, no tag	1-396, no tag	1-396, no tag	FtsA: 1-396, no tag; FtsN: 1-29, no tag
Crystallisation (sample; crystallisation condition)	7 mg/ml, 2 mM ATP; 20 % w/v PEG 4K, 0.175 M $(NH_4)_2SO_4$, 5 mM $Ni(II)Cl_2$, 0.1 M Tris/HAc pH 7.5, 0.017 M MES/NaOH pH 5.9	7 mg/ml, 2 mM ATP; 1 M sodium succinate pH 7.0, 0.1 M bis-tris-propane pH 7.0	5 mg/ml, 2 mM ATP; 25.236 % v/v PEG 400, 2 % v/v 2-propanol, 0.17 M $MgCl_2$, 0.04 M $CaAc_2$, 0.08 M Tris/HCl pH 8.5, 0.02 M MES/NaOH pH 6.0	VmFtsA: 3 mg/ml, VmFtsN$^{1-29}$: 0.353 mM (5x molar excess), 2 mM ATP; 21.645 % v/v PEG 600, 0.125 M $MgCl_2$, 0.1 M Tris/HCl pH 8.5
Cryoprotectant	20 % w/v PEG 4K, 0.262 M $(NH_4)_2SO_4$, 5 mM $Ni(II)Cl_2$, 0.1 M Tris/HAc pH 7.5, 0.011 M MES/NaOH pH 5.9, 25 % glycerol	reservoir solution + 25 % glycerol	2mM ATP, 31.545 % v/v PEG 400, 0.212 M $MgCl_2$, 0.1 M Tris/HCl pH 8.5, 0.1 M KCl, 5 mM TCEP, 20 % glycerol	0.353 mM VmFtsN$^{1-29}$, 2 mM ATP, 22.518 % v/v PEG 600, 0.129 M $MgCl_2$, 0.1 M Tris/HCl pH 8.5, 0.1 M KCl, 5 mM TCEP, 20 % glycerol, 2 mM EMTS (soaked for 1h)[4]
**Method**				
Method	crystallography	crystallography	crystallography	crystallography
Phasing	MR<br>model 3WQT without IC domain, which later was added manually	MR<br>model 7Q6D	MR<br>model 7Q6D	MR<br>model 7Q6F, then dimer from first Phaser run
**Data collection**				
Beamline	DLS I04	DLS I04	DLS I03	DLS I04
Wavelength (Å)	0.97948	1.00915	0.97625	1.00915
**Crystal**				
Space / point group	$P2_1$	C2	$P2_1$	$P2_1$
Cell (Å; °)	51.1, 64.2, 65.3; 96.1	142.2, 70.7, 50.8; 101.0	48.6, 109.8, 92.6; 102.1	98.8, 119.9, 330.9; 93.81
**Data**				
Resolution (Å)	2.8	2.8	3.3	3.6
Completeness (%)[1]	99.8 (99.9)	99.9 (99.9)	99.4 (99.0)	100.0 (100.0)
Multiplicity[1]	3.4 (3.4)	3.4 (3.0)	6.9 (6.4)	6.9 (7.1)
(I) / σ(I)[1]	6.8 (1.4)	4.4 (1.1)	5.9 (2.2)	8.6 (1.8)
R_{merge}[1]	0.142 (0.863)	0.257 (0.857)	0.297 (1.309)	0.163 (1.197)
R_{pim}[1]	0.091 (0.552)	0.165 (0.584)	0.120 (0.544)	0.067 (0.489)
CC1/2	0.991 (0.659)	0.796 (0.636)	0.978 (0.954)	0.997 (0.983)
**Refinement**				
R_{work} / R_{free}[2]	0.217 / 0.269	0.218 / 0.260	0.230 / 0.269	0.241 / 0.287
Models	1 FtsA monomer/ASU:<br>A: 6-388<br>1 ATP, 1 Mg, 3 Ni, no waters	1 FtsA monomer/ASU:<br>A: 5-392<br>1 ADP, 1 Mg, no waters	2 FtsA monomers/ASU:<br>A: 8-389<br>B: 8-389<br>2 ATP, 2 Mg, no waters	16 FtsA monomers and modelled backbones for 2 FtsN$^{1-29}$ monomers/ASU:<br>FtsA: A, C, E-P: 8-389, B: 7-389, D: 8-269, 290-389<br>FtsN$^{1-29}$: X-Y: unknown polyAla1-8 (refined as M1-R8)<br>NCS_groups: [**A**, C, E, G, I, K, M, O], [**B**, D, F, H, J, L, N, P], [**Y**, X]<br>(FtsN$^{1-29}$ density: strong: A, C; weak: E, G, I, K, M, O; none: B, D, F, H, J, L, N, P)<br>16 ATP, 16 Mg, no waters
Bond length rmsd (Å)	0.003	0.002	0.002	0.003
Bond angle rmsd (°)	0.511	0.538	0.504	0.657
Favoured (%)[3]	99.1	99.7	99.7	99.4
Disallowed (%)[3]	0.0	0.0	0.0	0.0
MOLPROBITY score	99th percentile	100th percentile	100th percentile	100th percentile
**PDB IDs**	7Q6D	7Q6G	7Q6F	7Q6I

[1] Values in parentheses refer to the highest recorded resolution shell. [2] 5% of reflections were randomly selected before refinement. [3] Percentage of residues in the Ramachandran plot (CCP4 PROCHECK: "most favoured" and "additionally allowed" added together). [4] Data collection was optimised for a native dataset, not SAD. Some density for mercury is however observed at C347 for chains A-F, H, J-L, N, P. The mercury density was used to verify the solution obtained by molecular replacement.

Supplementary Table T2. FtsN suppressor and ZipA suppressor and filament architecture mutants.

FtsA mutation	Description
G50E	Isolated as a suppressor of a thermosensitive FtsA bearing an ATP-binding pocket defect (S195P)[1], suppresses a thermosensitive *zipA1* (ts) allele[1], forms short, straight double filaments on lipid monolayer[2]
E124A	Isolated as a spontaneous suppressor of a Δ*ftsN* strain bearing a thermosensitive, *ftsN*-containing plasmid after long-term *ftsN* depletion[3], rescues Δ*ftsN* strain when overexpressed[4], suppresses Δ*zipA*[3]
Y139D	Isolated as a suppressor of a thermosensitive FtsA bearing an ATP-binding pocket defect (S195P)[1], suppresses a thermosensitive *zipA1* (ts) allele[1], forms “mini-rings”, arcs and straight filaments on lipid monolayer[2]
I143L	First isolated as a suppressor of a $ftsQ^{V92D}$ allele encoding a FtsQ variant defective in divisome localisation[5], suppresses Δ*zipA*[5], also isolated as a suppressor of the non-functional $gfp\text{-}ftsN^{Y85W}$ allele in a Δ*ftsN* strain bearing a thermosensitive, *ftsN*-containing plasmid[4], rescues Δ*ftsN* strain when overexpressed[4]
T249M	Isolated as a suppressor of a thermosensitive FtsA bearing an ATP-binding pocket defect (S195P)[1], suppresses a thermosensitive *zipA1* (ts) allele[1], forms mostly arcs on lipid monolayer[2]
R286W/FtsA*	Isolated as a suppressor of Δ*zipA* strain bearing a thermosensitive, *zipA*-containing plasmid, forms mostly arcs on lipid monolayer[2], weakly bypasses Δ*ftsN*[6]

Supplementary Table T3. Plasmids.

ID	Plasmid	Features	Source
pFB483	pCONEX-Gate4 CRISPR(*ftsW secM*) *ccdB*	Shuttle plasmid for targeting the *ftsA* locus ranging from *ftsW* to *secM* (*Bsa*I acceptor)	This study
pJF146	RK24 *lux apR bsd*	Conjugative plasmid, NCBI ID: MN927219.1	[7]
pKW20	Para *lambda-red cas9 tetR tracrRNA*	REXER helper plasmid, NCBI ID: MN927219.1	[8]
pTN_AN_001	pTXB1(C12H) EcFtsA	Expression plasmid for EcFtsA-intein-CBD-12H under T7 promoter	This study
pTN_AN_003	pTXB1(C12H) EcFtsA^{E124A}	Expression plasmid for EcFtsA^{E124A}-intein-CBD-12H under T7 promoter	This study
pTN_AN_004	pTXB1(C12H) EcFtsA^{I143L}	Expression plasmid for EcFtsA^{I143L}-intein-CBD-12H under T7 promoter	This study
pTN_AN_005	pTXB1(C12H) EcFtsA^{R286W}/EcFtsA*	Expression plasmid for EcFtsA^{R286W}/EcFtsA*-intein-CBD-12H under T7 promoter	This study
pTN_AN_022	pOPINS EcFtsA$^{1\text{-}405}$	Expression plasmid for 6H-SUMO-EcFtsA$^{1\text{-}405}$ under T7 promoter	This study
pTN_AN_024	pTXB1(C12H) EcFtsA^{G50E}	Expression plasmid for EcFtsA^{G50E}-intein-CBD-12H under T7 promoter	This study
pTN_AN_050	pOPINS XpFtsA$^{1\text{-}396}$	Expression plasmid for 6H-SUMO-XpFtsA$^{1\text{-}396}$ under T7 promoter	This study
pTN_AN_052	pOPINS VmFtsA$^{1\text{-}396}$	Expression plasmid for 6H-SUMO-VmFtsA$^{1\text{-}396}$ under T7 promoter	This study
pTN_AN_057	pTXB1(C12H) VmFtsA	Expression plasmid for VmFtsA-intein-CBD-12H under T7 promoter	This study
pTN_AN_059	pTXB1(C12H) EcFtsA$^{M96E, R153D}$	Expression plasmid for EcFtsA$^{M96E, R153D}$-intein-CBD-12H under T7 promoter	This study
pTN_AN_069	pOPINSb VmFtsN$^{1\text{-}29}$-ENLYFQ	Expression plasmid for VmFtsN$^{1\text{-}29}$-TEV-lipoyl tag-6H under T7 promoter in the pOPINS backbone (6H-SUMO tag was removed)	This study
pTN_AN_071	pOPINSb Gly-Gly-VmFtsN$^{2\text{-}29}$	Expression plasmid for 6H-lipoyl tag-TEV-G-VmFtsN$^{2\text{-}29}$ under T7 promoter in the pOPINS backbone (6H-SUMO tag was removed)	This study
pTN_AN_901	pET-24b(+) 6H-TEV protease	Expression plasmid for 6H-TEV protease (PolG* catalytic domain) under *tac* promoter	Aricescu lab, MRC LMB
pTN_AN_902	pGEX-6p-1 GST-SENP1	Expression plasmid for GST-SENP1 (C-terminal catalytic domain) under *tac* promoter	[9]

Supplementary Table T4. Protein sequences.

EcFtsA: *Escherichia coli* MG1655 FtsA, NCBI ID: AMK99051.1

EcFtsN: *Escherichia coli* MG1655 FtsA, NCBI ID: NP_418368.1

VmFtsA: *Vibrio maritimus* FtsA, NCBI ID: WP_042501243.1

VmFtsN: *Vibrio maritimus* FtsA, NCBI ID: WP_042502685.1

XpFtsA: *Xenorhabdus poinarii* G6 FtsA, NCBI ID: WP_045957782.1

Cleavage sites are marked with "///", with the target protein sequence after cleavage highlighted in bold. Mutations from wildtype are underlined.

EcFtsA from EcFtsA-intein-CBD-12H (pTN_AN_001)

MIKATDRKLVVGLEIGTAKVAALVGEVLPDGMVNIIGVGSCPSRGMDKGGVNDLESVVKCVQRAIDQAELMAD
CQISSVYLALSGKHISCQNEIGMVPISEEEVTQEDVENVVHTAKSVRVRDEHRVLHVIPQEYAIDYQEGIKNPVGL
SGVRMQAKVHLITCHNDMAKNIVKAVERCGLKVDQLIFAGLASSYSVLTEDERELGVCVVDIGGGTMDIAVYTG
GALRHTKVIPYAGNVVTSDIAYAFGTPPSDAEAIKVRHGCALGSIVGKDESVEVPSVGGRPPRSLQRQTLAEVIE
PRYTELLNLVNEEILQLQEKLRQQGVKHHLAAGIVLTGGAAQIEGLAACAQRVFHTQVRIGAPLNITGLTDYAQE
PYYSTAVGLLHYGKESHLNGEAEVEKRVTASVGSWIKRLNSWLRKEF///CITGDALVALPEGESVRIADIVPGAR
PNSDNAIDLKVLDRHGNPVLADRLFHSGEHPVYTVRTVEGLRVTGTANHPLLCLVDVAGVPTLLWKLIDEIKPGDY
AVIQRSAFSVDCAGFARGKPEFAPTTYTVGVPGLVRFLEAHHRDPDAQAIADELTDGRFYYAKVASVTDAGVQP
VYSLRVDTADHAFITNGFVSHATGLTGLNSGLTTNPGVSAWQVNTAYTAGQLVTYNGKTYKCLQPHTSLAGWEP
SNVPALWQLQGSSGGHHHHHHHHHHHH

EcFtsA^{E124A} from EcFtsA^{E124A}-intein-CBD-12H (pTN_AN_003)

MIKATDRKLVVGLEIGTAKVAALVGEVLPDGMVNIIGVGSCPSRGMDKGGVNDLESVVKCVQRAIDQAELMAD
CQISSVYLALSGKHISCQNEIGMVPISEEEVTQEDVENVVHTAKSVRVRDAHRVLHVIPQEYAIDYQEGIKNPVGL
SGVRMQAKVHLITCHNDMAKNIVKAVERCGLKVDQLIFAGLASSYSVLTEDERELGVCVVDIGGGTMDIAVYTG
GALRHTKVIPYAGNVVTSDIAYAFGTPPSDAEAIKVRHGCALGSIVGKDESVEVPSVGGRPPRSLQRQTLAEVIE
PRYTELLNLVNEEILQLQEKLRQQGVKHHLAAGIVLTGGAAQIEGLAACAQRVFHTQVRIGAPLNITGLTDYAQE
PYYSTAVGLLHYGKESHLNGEAEVEKRVTASVGSWIKRLNSWLRKEF///CITGDALVALPEGESVRIADIVPGAR
PNSDNAIDLKVLDRHGNPVLADRLFHSGEHPVYTVRTVEGLRVTGTANHPLLCLVDVAGVPTLLWKLIDEIKPGDY
AVIQRSAFSVDCAGFARGKPEFAPTTYTVGVPGLVRFLEAHHRDPDAQAIADELTDGRFYYAKVASVTDAGVQP
VYSLRVDTADHAFITNGFVSHATGLTGLNSGLTTNPGVSAWQVNTAYTAGQLVTYNGKTYKCLQPHTSLAGWEP
SNVPALWQLQGSSGGHHHHHHHHHHHH

EcFtsA^{I143L} from EcFtsA^{I143L}-intein-CBD-12H (pTN_AN_004)

MIKATDRKLVVGLEIGTAKVAALVGEVLPDGMVNIIGVGSCPSRGMDKGGVNDLESVVKCVQRAIDQAELMAD
CQISSVYLALSGKHISCQNEIGMVPISEEEVTQEDVENVVHTAKSVRVRDEHRVLHVIPQEYAIDYQEGLKNPVG
LSGVRMQAKVHLITCHNDMAKNIVKAVERCGLKVDQLIFAGLASSYSVLTEDERELGVCVVDIGGGTMDIAVYT
GGALRHTKVIPYAGNVVTSDIAYAFGTPPSDAEAIKVRHGCALGSIVGKDESVEVPSVGGRPPRSLQRQTLAEVI
EPRYTELLNLVNEEILQLQEKLRQQGVKHHLAAGIVLTGGAAQIEGLAACAQRVFHTQVRIGAPLNITGLTDYAQ
EPYYSTAVGLLHYGKESHLNGEAEVEKRVTASVGSWIKRLNSWLRKEF///CITGDALVALPEGESVRIADIVPGA
RPNSDNAIDLKVLDRHGNPVLADRLFHSGEHPVYTVRTVEGLRVTGTANHPLLCLVDVAGVPTLLWKLIDEIKPGD
YAVIQRSAFSVDCAGFARGKPEFAPTTYTVGVPGLVRFLEAHHRDPDAQAIADELTDGRFYYAKVASVTDAGVQ

PVYSLRVDTADHAFITNGFVSHATGLTGLNSGLTTNPGVSAWQVNTAYTAGQLVTYNGKTYKCLQPHTSLAGWE
PSNVPALWQLQGSSGGHHHHHHHHHHHH

EcFtsA$^{\mathrm{R286W}}$/EcFtsA* from EcFtsA$^{\mathrm{R286W}}$-intein-CBD-12H (pTN_AN_005)

MIKATDRKLVVGLEIGTAKVAALVGEVLPDGMVNIIGVGSCPSRGMDKGGVNDLESVVKCVQRAIDQAELMAD
CQISSVYLALSGKHISCQNEIGMVPISEEEVTQEDVENVVHTAKSVRVRDEHRVLHVIPQEYAIDYQEGIKNPVGL
SGVRMQAKVHLITCHNDMAKNIVKAVERCGLKVDQLIFAGLASSYSVLTEDERELGVCVVDIGGGTMDIAVYTG
GALRHTKVIPYAGNVVTSDIAYAFGTPPSDAEAIKVRHGCALGSIVGKDESVEVPSVGGRPPWSLQRQTLAEVI
EPRYTELLNLVNEEILQLQEKLRQQGVKHHLAAGIVLTGGAAQIEGLAACAQRVFHTQVRIGAPLNITGLTDYAQ
EPYYSTAVGLLHYGKESHLNGEAEVEKRVTASVGSWIKRLNSWLRKEF///CITGDALVALPEGESVRIADIVPGA
RPNSDNAIDLKVLDRHGNPVLADRLFHSGEHPVYTVRTVEGLRVTGTANHPLLCLVDVAGVPTLLWKLIDEIKPGD
YAVIQRSAFSVDCAGFARGKPEFAPTTYTVGVPGLVRFLEAHHRDPDAQAIADELTDGRFYYAKVASVTDAGVQ
PVYSLRVDTADHAFITNGFVSHATGLTGLNSGLTTNPGVSAWQVNTAYTAGQLVTYNGKTYKCLQPHTSLAGWE
PSNVPALWQLQGSSGGHHHHHHHHHHHH

EcFtsA$^{\mathrm{G50E}}$ from EcFtsA$^{\mathrm{G50E}}$-intein-CBD-12H (pTN_AN_024)

MIKATDRKLVVGLEIGTAKVAALVGEVLPDGMVNIIGVGSCPSRGMDKGEVNDLESVVKCVQRAIDQAELMAD
CQISSVYLALSGKHISCQNEIGMVPISEEEVTQEDVENVVHTAKSVRVRDEHRVLHVIPQEYAIDYQEGIKNPVGL
SGVRMQAKVHLITCHNDMAKNIVKAVERCGLKVDQLIFAGLASSYSVLTEDERELGVCVVDIGGGTMDIAVYTG
GALRHTKVIPYAGNVVTSDIAYAFGTPPSDAEAIKVRHGCALGSIVGKDESVEVPSVGGRPPRSLQRQTLAEVIE
PRYTELLNLVNEEILQLQEKLRQQGVKHHLAAGIVLTGGAAQIEGLAACAQRVFHTQVRIGAPLNITGLTDYAQE
PYYSTAVGLLHYGKESHLNGEAEVEKRVTASVGSWIKRLNSWLRKEF///CITGDALVALPEGESVRIADIVPGAR
PNSDNAIDLKVLDRHGNPVLADRLFHSGEHPVYTVRTVEGLRVTGTANHPLLCLVDVAGVPTLLWKLIDEIKPGDY
AVIQRSAFSVDCAGFARGKPEFAPTTYTVGVPGLVRFLEAHHRDPDAQAIADELTDGRFYYAKVASVTDAGVQP
VYSLRVDTADHAFITNGFVSHATGLTGLNSGLTTNPGVSAWQVNTAYTAGQLVTYNGKTYKCLQPHTSLAGWEP
SNVPALWQLQGSSGGHHHHHHHHHHHH

VmFtsA from VmFtsA-intein-CBD-12H (pTN_AN_057)

MTKTTDDNIIVGLDIGTATVSALVGEVLPDGQVNIIGAGSSPSRGMDKGGVNDLESVVKSVQRAVDQAELMAEC
QISSVFISLSGKHIASRIEKGMGTISEEEVSQDDMDRAIHTAKSIKIGDEQRILHVIPQEFTIDYQEGIKNPLGLSGV
RMEVSVHLISCHNDMARNIIKAVERCGLKVEQLVFSGLASSNAVITEDERELGVCVVDIGAGTMDISIWTGGALR
HTEVFSYAGNAVTSDIAFAFGTPLSDAEEIKVKYGCALSELVSKDDTVNVPSVGGRPSRSLQRQTLAEVIEPRYT
ELMGLVNQTIDNVQAKLRENGVKHHLAAGVVLTGGAAQIEGVVECAERVFRNQVRVGKPLEVSGLTDYVKEPY
HSTAVGLLHYARDSQDNDDNDYNEPKRQSVSTIFGKLRNWIQKEF///CITGDALVALPEGESVRIADIVPGARPN
SDNAIDLKVLDRHGNPVLADRLFHSGEHPVYTVRTVEGLRVTGTANHPLLCLVDVAGVPTLLWKLIDEIKPGDYAV
IQRSAFSVDCAGFARGKPEFAPTTYTVGVPGLVRFLEAHHRDPDAQAIADELTDGRFYYAKVASVTDAGVQPVY
SLLVDTADHAFITNGFVSHATGLTGLNSGLTTNPGVSAWQVNTAYTAGQLVTYNGKTYKCLQPHTSLAGWEPSN
VPALWQLQGSSGGHHHHHHHHHHHH

EcFtsA$^{\mathrm{M96E,\ R153D}}$ from EcFtsA$^{\mathrm{M96E,\ R153D}}$-intein-CBD-12H (pTN_AN_059)

MIKATDRKLVVGLEIGTAKVAALVGEVLPDGMVNIIGVGSCPSRGMDKGGVNDLESVVKCVQRAIDQAELMAD
CQISSVYLALSGKHISCQNEIGEVPISEEEVTQEDVENVVHTAKSVRVRDEHRVLHVIPQEYAIDYQEGIKNPVGL
SGVDMQAKVHLITCHNDMAKNIVKAVERCGLKVDQLIFAGLASSYSVLTEDERELGVCVVDIGGGTMDIAVYTG
GALRHTKVIPYAGNVVTSDIAYAFGTPPSDAEAIKVRHGCALGSIVGKDESVEVPSVGGRPPRSLQRQTLAEVIE
PRYTELLNLVNEEILQLQEKLRQQGVKHHLAAGIVLTGGAAQIEGLAACAQRVFHTQVRIGAPLNITGLTDYAQE
PYYSTAVGLLHYGKESHLNGEAEVEKRVTASVGSWIKRLNSWLRKEF///CITGDALVALPEGESVRIADIVPGAR
PNSDNAIDLKVLDRHGNPVLADRLFHSGEHPVYTVRTVEGLRVTGTANHPLLCLVDVAGVPTLLWKLIDEIKPGDY
AVIQRSAFSVDCAGFARGKPEFAPTTYTVGVPGLVRFLEAHHRDPDAQAIADELTDGRFYYAKVASVTDAGVQP
VYSLRVDTADHAFITNGFVSHATGLTGLNSGLTTNPGVSAWQVNTAYTAGQLVTYNGKTYKCLQPHTSLAGWEP
SNVPALWQLQGSSGGHHHHHHHHHHHH

EcFtsA$^{\mathrm{1\text{-}405}}$ from 6H-SUMO-EcFtsA$^{\mathrm{1\text{-}405}}$ (pTN_AN_022)

MGSSHHHHHHGSDSEVNQEAKPEVKPEVKPETHINLKVSDGSSEIFFKIKKTTPLRRLMEAFAKRQGKEMDSLRF
LYDGIRIQADQTPEDLDMEDNDIIEAHREQIGG///**MIKATDRKLVVGLEIGTAKVAALVGEVLPDGMVNIIGVGSCP**
SRGMDKGGVNDLESVVKCVQRAIDQAELMADCQISSVYLALSGKHISCQNEIGMVPISEEEVTQEDVENVVHTA
KSVRVRDEHRVLHVIPQEYAIDYQEGIKNPVGLSGVRMQAKVHLITCHNDMAKNIVKAVERCGLKVDQLIFAGL
ASSYSVLTEDERELGVCVVDIGGGTMDIAVYTGGALRHTKVIPYAGNVVTSDIAYAFGTPPSDAEAIKVRHGCAL
GSIVGKDESVEVPSVGGRPPRSLQRQTLAEVIEPRYTELLNLVNEEILQLQEKLRQQGVKHHLAAGIVLTGGAA
QIEGLAACAQRVFHTQVRIGAPLNITGLTDYAQEPYYSTAVGLLHYGKESHLNGEAEVEKRVTASV

XpFtsA$^{1-396}$ from 6H-SUMO-XpFtsA$^{1-396}$ (pTN_AN_050)

MGSSHHHHHHGSDSEVNQEAKPEVKPEVKPETHINLKVSDGSSEIFFKIKKTTPLRRLMEAFAKRQGKEMDSLRF
LYDGIRIQADQTPEDLDMEDNDIIEAHREQIGG///**MIKSTDRKLVVGLEIGTAKVSALVGEILPDGMVNIIGVGNCPS**
RGMDKGGVNDLESVVKCVQRAIDQAELMADCQISSVYLALSGKHISCQNEIGMVPVSEEEVTQDDVDSVVHTA
KSVRVRDEHRILHVIPQEYAIDYQEGIKNPVGLSGVRMQAKVHLITCHNDMAKNIVKAVERCGLKVDQLIFAGLA
ASYAVLTEDERELGVCVVDIGGGTMDVAVYTGGALRHTKVIPYAGNVVTSDIAYAFGTPPSDAETIKVRHGCAL
GSIVSKDESVEVPSVGGRPPRSLQRQTLAEVIEPRYTELLNLVNDEILRLQEQLRQQGVKHHLAAGIVLTGGGA
QIDGLAECAQRVFHTQVRIGRPLNITGLTDYVQAPCYSTAVGLLHYGKESHLGGDSD

VmFtsA$^{1-396}$ from 6H-SUMO-VmFtsA$^{1-396}$ (pTN_AN_052)

MGSSHHHHHHGSDSEVNQEAKPEVKPEVKPETHINLKVSDGSSEIFFKIKKTTPLRRLMEAFAKRQGKEMDSLRF
LYDGIRIQADQTPEDLDMEDNDIIEAHREQIGG///**MTKTTDDNIIVGLDIGTATVSALVGEVLPDGQVNIIGAGSSPS**
RGMDKGGVNDLESVVKSVQRAVDQAELMAECQISSVFISLSGKHIASRIEKGMGTISEEEVSQDDMDRAIHTAK
SIKIGDEQRILHVIPQEFTIDYQEGIKNPLGLSGVRMEVSVHLISCHNDMARNIIKAVERCGLKVEQLVFSGLASSN
AVITEDERELGVCVVDIGAGTMDISIWTGGALRHTEVFSYAGNAVTSDIAFAFGTPLSDAEEIKVKYGCALSELVS
KDDTVNVPSVGGRPSRSLQRQTLAEVIEPRYTELMGLVNQTIDNVQAKLRENGVKHHLAAGVVLTGGAAQIEG
VVECAERVFRNQVRVGKPLEVSGLTDYVKEPYHSTAVGLLHYARDSQDNDDND

VmFtsN$^{1-29}$-ENLYFQ from VmFtsN$^{1-29}$-TEV-lipoyl tag-6H (pTN_AN_069)

M‡///**ANRDYVRRGKGTSRRPAKKKTSGKKPWRENLYFQ**///GGSSGAFEFKLPDIGEGIHEGEIVKWFVKPGDEV
NEDDVLCEVQNDKAVVEIPSPVKGKVLEILVPEGTVATVGQTLITLDAPGYENMTTGSDTGSHHHHHH
‡N-terminal methionine was cleaved off *in vivo* according to ESI-TOF mass spectrometry.

Gly-Gly-VmFtsN$^{2-29}$ from 6H-lipoyl tag-TEV-G-VmFtsN$^{2-29}$ (pTN_AN_071)

MGSSHHHHHHSGAFEFKLPDIGEGIHEGEIVKWFVKPGDEVNEDDVLCEVQNDKAVVEIPSPVKGKVLEILVPEG
TVATVGQTLITLDAPGYENMTTGSDTGSENLYFQ///**GGANRDYVRRGKGTSRRPAKKKTSGKKPWR**

Supplementary Table T5. FtsN peptide sequences.

Peptide	Sequence	MW (Da)
EcFtsN$^{1-12}$	MAQRD YVRRS QP	1506.70
EcFtsN$^{1-22}$	MAQRD YVRRS QPAPS RRKKS TS	2605.96
EcFtsN$^{11-32}$	QPAPS RRKKS TSRKK QRNLP AV	2533.97
EcFtsN$^{4-26}$	RD YVRRS QPAPS RRKKS TSRKK Q	2816.23
EcFtsN$^{1-32}$	MAQRD YVRRS QPAPS RRKKS TSRKK QRNLP AV	3797.41
EcFtsN$^{1-32,\ D5N}$	MAQR**N** YVRRS QPAPS RRKKS TSRKK QRNLP AV	3796.42
EcFtsN$^{1-32,\ \Delta RK1}$	MAQRD YV**AA**S QPAPS RRKKS TSRKK QRNLP AV	3627.19
EcFtsN$^{1-32;\ \Delta RK2}$	MAQRD YVRRS QPAPS **AASA**S TSRKK QRNLP AV	3529.00
EcFtsN$^{1-32,\ \Delta RK3}$	MAQRD YVRRS QPAPS RRKKS TS**ASA** QRNLP AV	3614.11
EcFtsN$^{1-32,\ \Delta RK2,3}$	MAQRD YVRRS QPAPS **AASA**S TS**ASA** QRNLP AV	3345.70
EcFtsN$^{1-33,\ scrambled\ *}$	SARRK NKRRV RQVRK TAPPQ QDSSP LKAMS RYS	3884.48
EcFtsN$^{1-32}$-C	MAQRD YVRRS QPAPS RRKKS TSRKK QRNLP AV**C**	3900.55
VmFtsN$^{1-29}$	MANRD YVRRG KGTSR RPAKK KTSGK KPWR	3417.00
VmFtsN$^{1-29}$-C	MANRD YVRRG KGTSR RPAKK KTSGK KPWR**C**	3520.14

* Shortened version (tags removed) of the scrambled FtsN peptide control used by Baranova and co-workers[10]

Supplementary Table T6. *E. coli* strains.

Strain	Genotype	Source	NGS*
E. coli C41(DE3)	F− *ompT hsdSB (rB− mB−) gal dcm* (DE3); BL21(DE3) derivative	Lucigen or Sigma	No
E. coli MAX Efficiency DH5α	F− φ80*lac*ZΔM15 Δ(*lac*ZYA-argF) U169 *rec*A1 *end*A1 *hsd*R17 (rk−, mk+) *pho*A *sup*E44 *λ*− *thi*−1 *gyr*A96 *rel*A1	ThermoFisher	No
MDS42	MG1655, Δ*endA*, Δ*fhuABCD*, deletion of IS elements, prophages etc	Chin lab, MRC LMB [11]	No
MG1655	F− *λ*− *rph*−1 *fnr*+	DSMZ (DSM 18039)	*rclC*^{G169C}
SFB123	MG1655, *lpxC::pheS*^{T251A,A294G}*-hygR*, pkW20 Para *lambda-red cas9 tetR tracrRNA*	This study	*rclC*^{G169C}, *ppiD*^{D134G}
SFB143	MDS42, Δ*thiF*, pJF146 RK24 *lux apR bsd*	This study	No
SFB148	MG1655, *lpxC::neoR*, pkW20 Para *lambda-red cas9 tetR tracrRNA*	This study	No
sTN001	MG1655, *ftsA*$^{269\text{-}3x\ HA\text{-}tag\text{-}270(SW)}$, *lpxC::neoR*	This study	*rclC*^{G169C}, *ppiD*^{D134G}
sTN003	MG1655, *ftsA*$^{P98C,\ 269\text{-}3x\ HA\text{-}tag\text{-}270(SW)}$, *lpxC::neoR*	This study	*rclC*^{G169C}, *ppiD*^{D134G}
sTN005	MG1655, *ftsA*$^{P98C,\ S118C,\ 269\text{-}3x\ HA\text{-}tag\text{-}270(SW)}$, *lpxC::neoR*	This study	*rclC*^{G169C}, *ppiD*^{D134G}
sTN008	MG1655, *ftsA*$^{S118C,\ 269\text{-}3x\ HA\text{-}tag\text{-}270(SW)}$, *lpxC::neoR*	This study	*rclC*^{G169C}, *ppiD*^{D134G}
sTN010	MG1655, *ftsA*$^{E199C,\ S252C,\ 269\text{-}3x\ HA\text{-}tag\text{-}270(SW)}$, *lpxC::neoR*	This study	*rclC*^{G169C}, *ppiD*^{D134G}
sTN011	MG1655, *ftsA*$^{E199C,\ 269\text{-}3x\ HA\text{-}tag\text{-}270(SW)}$, *lpxC::neoR*	This study	*rclC*^{G169C}, *ppiD*^{D134G}
sTN014	MG1655, *ftsA*$^{S252C,\ 269\text{-}3x\ HA\text{-}tag\text{-}270(SW)}$, *lpxC::neoR*	This study	*rclC*^{G169C}, *ppiD*^{D134G}
sTN015	MG1655, *ftsA*$^{Q155C,\ 269\text{-}3x\ HA\text{-}tag\text{-}270(SW)}$, *lpxC::neoR*	This study	No
sTN017	MG1655, *ftsA*$^{D123C,\ 269\text{-}3x\ HA\text{-}tag\text{-}270(SW)}$, *lpxC::neoR*	This study	No

* Intragenic, missense mutations only. For more details see Supplementary Data D4.

SUPPLEMENTARY REFERENCES

1 Herricks, J. R., Nguyen, D. & Margolin, W. A thermosensitive defect in the ATP binding pocket of FtsA can be suppressed by allosteric changes in the dimer interface. *Mol Microbiol* **94**, 713-727, doi:10.1111/mmi.12790 (2014).

2 Schoenemann, K. M. *et al.* Gain-of-function variants of FtsA form diverse oligomeric structures on lipids and enhance FtsZ protofilament bundling. *Mol Microbiol*, doi:10.1111/mmi.14069 (2018).

3 Bernard, C. S., Sadasivam, M., Shiomi, D. & Margolin, W. An altered FtsA can compensate for the loss of essential cell division protein FtsN in Escherichia coli. *Mol Microbiol* **64**, 1289-1305, doi:10.1111/j.1365-2958.2007.05738.x (2007).

4 Liu, B., Persons, L., Lee, L. & de Boer, P. A. Roles for both FtsA and the FtsBLQ subcomplex in FtsN-stimulated cell constriction in Escherichia coli. *Mol Microbiol* **95**, 945-970, doi:10.1111/mmi.12906 (2015).

5 Goehring, N. W., Petrovska, I., Boyd, D. & Beckwith, J. Mutants, suppressors, and wrinkled colonies: mutant alleles of the cell division gene ftsQ point to functional domains in FtsQ and a role for domain 1C of FtsA in divisome assembly. *J Bacteriol* **189**, 633-645, doi:10.1128/JB.00991-06 (2007).

6 Park, K. T., Pichoff, S., Du, S. & Lutkenhaus, J. FtsA acts through FtsW to promote cell wall synthesis during cell division in Escherichia coli. *Proc Natl Acad Sci U S A* **118**, doi:10.1073/pnas.2107210118 (2021).

7 Fredens, J. *et al.* Total synthesis of Escherichia coli with a recoded genome. *Nature* **569**, 514-518, doi:10.1038/s41586-019-1192-5 (2019).

8 Wang, K. *et al.* Defining synonymous codon compression schemes by genome recoding. *Nature* **539**, 59-64, doi:10.1038/nature20124 (2016).

9 van den Ent, F., Izore, T., Bharat, T. A., Johnson, C. M. & Löwe, J. Bacterial actin MreB forms antiparallel double filaments. *Elife* **3**, e02634, doi:10.7554/eLife.02634 (2014).

10 Baranova, N. *et al.* Diffusion and capture permits dynamic coupling between treadmilling FtsZ filaments and cell division proteins. *Nat Microbiol*, doi:10.1038/s41564-019-0657-5 (2020).

11 Posfai, G. *et al.* Emergent properties of reduced-genome Escherichia coli. *Science* **312**, 1044-1046, doi:10.1126/science.1126439 (2006).
