# Supplementary material for: Bacterial divisome protein FtsA forms curved antiparallel double filaments upon binding FtsN
Source: Nat Microbiol. Author manuscript; Available in PMC 2022 Dec 14. (PMC7613929; doi:10.1038/s41564-022-01206-9)
Supplement: InventoryOfSupplementaryMaterial PMC Europe [file EMS151223-supplement-InventoryOfSupplementaryMaterial_PMC_Europe.docx]

INVENTORY OF SUPPLEMENTARY MATERIAL

**Bacterial divisome protein FtsA forms curved antiparallel double filaments upon binding FtsN**

Tim Nierhaus^1^, Stephen H McLaughlin^1^, Frank Bürmann^1^, Danguole Kureisaite-Ciziene^1^, Sarah L Maslen^1,2^, J Mark Skehel^1,2^, Conny WH Yu^1^, Stefan MV Freund^1^, Louise FH Funke^1^, Jason W Chin^1^, Jan Löwe^1^*

^1^ MRC Laboratory of Molecular Biology, Cambridge, UK.

^2^ Present address: The Francis Crick Institute, London, UK.

*Corresponding author: Jan Löwe, MRC Laboratory of Molecular Biology, Cambridge Biomedical Campus, Francis Crick Avenue, Cambridge CB2 0QH, UK, email: [jyl@mrc-lmb.cam.ac.uk](mailto:jyl@mrc-lmb.cam.ac.uk), phone: +44 (0)1223 267064

CONTENTS

Extended Data Figures 1-5 (Legends)

Supplementary Information (Legend)

Supplementary Movie M1 (Legend)

Supplementary Datasets D1-4 (Legends)

Source Data (Legends)

EXTENDED DATA FIGURES (LEGENDS)

Extended Data Figure 1. The position of the IC domain within the FtsA monomer varies.

**a**, Comparison of longitudinal filament contacts in FtsA crystal structures from *E. coli* (PDB 7Q6D), *X. poinarii* (PDB 7Q6G), *V. maritimus* (PDB 7Q6F) and *T. maritima* (PDB 4A2B). *E. coli* and *X. poinarii* FtsA form “loose” protofilaments with detached IIA and IIB domains (arrowhead). IIA and IIB domains are in close contact in the VmFtsA and TmFtsA structures, which form continuous filaments in the crystals. **b**, The IC domain of FtsA is flexible. Left: an arrow along the first principal axis of inertia of the IC domain (purple) can be used to indicate IC domain orientation. Right: FtsA structures in the PDB aligned on their IA, IIA and IIB domains, with arrows indicating the position of the IC domains, showing that the IC domain orientation is variable within the FtsA monomer. There is no correlation between IC domain position and species (different colours) or formation of continuous filaments in the crystals (^‡^). Principal axes of inertia were calculated using main chain atoms (N, CA, C) of IC domains. **c**, Comparison between the FtsA and MreB double filaments. Because the lateral interface is formed by the IC domain in the FtsA double filament, it is wider than the MreB double filament. The membrane‑proximal side of both double filaments is flat.

Extended Data Figure 2. VmFtsA forms antiparallel double filaments upon binding VmFtsN^1-29^.

**a**, Schematic overview of EcFtsN. **b**, SPR equilibrium response titration of VmFtsN^1‑29^ binding to immobilised VmFtsA^1‑396^. Binding affinity is about three‑fold lower than for the EcFtsA^1-405^-EcFtsN^1‑32^ interaction (Figure 2b). **c**, VmFtsA^1‑396^ titration into VmFtsN^1-29^-C-Atto 495. Data were fitted with a two‑step model, with transitions being indicative of FtsN binding and polymerisation (panel d). A representative quadruplicate is shown. K_d_s are given as mean ± SEM from five independent experiments. **d**, Weight-averaged sedimentation coefficients of a VmFtsA^1-396^ titration into VmFtsN^1-29^-C-Atto 495 by FDS-AUC shows that VmFtsN^1‑29^ is part of higher order FtsA polymers. Data were fitted to a two‑step model, recapitulating the FP data in panel c. **e**, Co‑pelleting assay of VmFtsN^1-29^ titrated into VmFtsA^1-396^ indicates that VmFtsN^1-29^ induces FtsA polymerisation. A representative SDS-PAGE gel is shown. Given are mean ± sd (black lines) of technical duplicates (white dots). P: pellet, S: supernatant. **f**, Negative stain electron micrographs of VmFtsA with and without VmFtsN^1-29^ on supported lipid monolayers. VmFtsA forms “mini-rings” in the absence of FtsN and double filaments at ten‑fold molar excess of VmFtsN^1-29^. Two independent grids were examined per condition. Scale bar, 50 nm, 20 nm (inset). **g**, Multiple sequence alignment of 245 FtsN sequences comprising cytoplasmic and transmembrane domains. EcFtsN^1‑32^ and VmFtsN^1-29^ sequences are highlighted in bold. **h**, Mapping of the FtsA-interacting region in EcFtsN^1-32^ using the lipid monolayer assay. EcFtsN^1-32^ mutants were in ten‑fold molar excess of FtsA. In contrast to EcFtsN^1‑32^, EcFtsN^1-32, D5N^ and a scrambled version of the EcFtsN^1‑32^ peptide^24^ did not induce FtsA double filaments. EcFtsN^4‑26^ and EcFtsN^1-32, ΔRK1^ led to formation of fewer double filaments. At least two independent grids were examined per condition. Scale bar, 20 nm. **i**, Summary of EcFtsN^1-32^ peptides. Mutations are highlighted in bold. Equilibrium dissociation constants (K^eq^_d_) are given as mean ± SEM (n ≥ 2 for each construct). For weak binders the maximum response was fixed during fitting, hence these are only approximate values as indicated by asterisks. ND: not determinable. The predominant higher order polymer observed in the monolayer assay is given in the “EM” column. Note that EcFtsN^4-26^ and EcFtsN^1‑32, ΔRK1^ still lead to formation of a few FtsA double filaments.

Extended Data Figure 3. Modelling of VmFtsN^1‑29^ binding to VmFtsA^1‑396^.

**a**, Comparison between the peptide-bound closed (coloured) and peptide-free open conformer (black) of FtsA in the VmFtsA^1-396^-VmFtsN^1-29^ co-crystal structure (PDB 7Q6I). The IC domain of the open conformer is rotated 13.8° downwards compared to the closed conformer, as determined by analysis with DynDom^38^. Consequently, the open conformation is likely incompatible with VmFtsN^1-29^ binding. The inset shows the position of open and closed conformers within the tetramer. **b**, Left: EcFtsA forms “mini-rings” on lipid monolayers as determined by cryo‑EM. Two independent grids were examined. Data was collected on one grid. Right: a computed 2D projection after expansion of a longitudinal dimer from the VmFtsA^1‑396^-VmFtsN^1-29^ co-crystal structure (PDB 7Q6I) is shown for comparison. The expanded longitudinal dimer does not form a closed ring but a helix. The comparison illustrates that FtsA’s IA domains are facing outwards. Scale bar, 20 nm. **c**, Comparison between the *V. maritimus* FtsA-FtsN and *Thermus thermophilus* PilM-PilN (PDB 2YCH) interaction sites^39^. Both binding sites are in the IA-IC interdomain cleft of FtsA and PilM but occupy distinct subspaces. FtsN predominantly contacts the IC domain of FtsA, whereas PilN binds closer to the IA domain of PilM. **d**, Stereo images of the FtsA-FtsN interaction site in the VmFtsA^1‑396^-VmFtsN^1-29^ co-crystal structure (PDB 7Q6I). Top: our preferred interpretation of the electron density corresponding to VmFtsN^1-29^, with residues M1-R8 modelled (purple). Side chains of FtsA residues in the interaction site are shown as sticks and polar contacts are marked with black, dashed lines. Bottom: electron density interpretation guided by the NMR data instead (panels e and f), with residues Y6-K11 of VmFtsN^1‑29^ modelled (purple). Electron density maps (grey) are shown at 1.2 sigma. **e**, ^1^H, ^15^N 2D-HSQC NMR spectrum of free Gly-Gly-VmFtsN^2-29^ (blue) and with equimolar amounts of FtsA added (orange). To follow VmFtsN numbering, the first glycine of Gly-Gly-VmFtsN^2-29^ is assigned as G0. **f**, Changes in relative peak intensity expressed as I_bound_/I_free_ with intensities normalised to I_R29_, which is assumed not to be involved in the VmFtsA-FtsN interaction.

Extended Data Figure 4. FtsA cysteine mutant strains: generation and absence of phenotype changes.

**a**, Workflow for REXER^73^-based strain construction. PCR products containing the 3x HA-tag or cysteine point mutations were inserted into a shuttle vector by Golden Gate assembly^78^. Assembled shuttle vectors were transformed into the donor strain, conjugated, and excised *in vivo* using Cas9. Targeting constructs contain homology regions for 𝜆‑Red mediated recombination into the target locus. Recombinants were selected for *neoR* and *tetR* markers and against the *pheS** marker. Strains were cured of the helper plasmid pKW20 by growth in absence of selection. **b**, Growth of strains containing single or double cysteine point mutations and a 3x HA-tag in the endogenous *ftsA* gene, and a kanamycin resistance cassette inserted after the *lpxC* gene. Parent strains and the original MG1655 strain are also shown. SW: sandwich fusion. **c**, Growth curves of the same strains in liquid LB medium. Plotted are traces of technical octuplicates (coloured) with mean (black). **d**, DIC images of the same strains in exponential phase (OD_600_ = 0.2-0.3) demonstrating the absence of elongated cells. Similar results were achieved in biological triplicate, of which one was imaged using DIC and two were imaged using phase contrast.

Extended Data Figure 5. Distances between endogenous and mutated cysteines in the FtsA double filament.

**a**, Positions of endogenous cysteines in EcFtsA (grey spheres) and of all cysteine mutations used for *in vivo* cysteine cross-linking (black sticks) (Figure 5a) are highlighted on the VmFtsA double filament structure (PDB 7Q6F). Dotted lines indicate intermolecular C_β_-C_β_ (putative cross-link) distances between selected cysteine mutations and the closest endogenous cysteine, the shortest distance being 15.9 Å (FtsA_i_ C163-FtsA_i*_ D123C). The inset highlights the interfaces in the VmFtsA double filament. SW: sandwich fusion. **b**, Single cysteine point mutations serve as controls for distance-independent intermolecular cross-linking because of the symmetry of the FtsA double filament, as illustrated on the example of P98C. The P98C mutation used for *in vivo* cysteine cross-linking is highlighted on the VmFtsA double filament structure (PDB 7Q6F). C_β_-C_β_ distances between intermolecular P98C mutations are indicated by dotted lines. The inset provides a comparison between experimentally sampled intermolecular C_β_-C_β_ distances by single cysteine point mutations (orange) and all intermolecular C_β_-C_β_ distances between amino acids P98, S118, E199 and S252 (blue). Single cysteine point mutations sample intermolecular distances similar to those between double cysteine mutations. Calculated intermolecular C_β_-C_β_ distances were rounded to one digit and duplicate values removed prior to plotting.

SUPPLEMENTARY INFORMATION (LEGEND)

Supplementary Information.

Supplementary Tables T1-6, Supplementary References.

SUPPLEMENTARY MOVIE (LEGEND)

Supplementary Movie M1.

Overview of the VmFtsA^1-396^-VmFtsN^1-29^ co-crystal structure (PDB 7Q6I). The 16 FtsA monomers are organised in short, antiparallel, and curved tetramers. Each protofilament contains one FtsA monomer in the open and closed conformation. Density for VmFtsN^1-29^ is only observed in the IA-IC interdomain cleft of closed conformers. The IC domain of open conformers is rotated downwards and is likely incompatible with VmFtsN^1-29^ binding.

SUPPLEMENTARY DATASETS (LEGENDS)

Supplementary Data D1.

Vector maps.

Supplementary Data D2.

Crystallographic models.

Supplementary Data D3.

Annotated genomic loci of *E. coli* strains.

Supplementary Data D4.

Summary of NGS analysis.

SOURCE DATA (LEGENDS)

Source Data Figure 2_numerical.

Numerical data for Figure 2b-e.

Source Data Figure 2_gel.

Unprocessed SDS-PAGE gels for Figure 2e.

Source Data Figure 5.

Unprocessed SDS-PAGE gels for Figure 5c, e.

Source Data Extended Data Figure 2_numerical.

Numerical data for Extended Data Figure 2b-e.

Source Data Extended Data Figure 2_gels.

Unprocessed SDS-PAGE gels for Extended Data Figure 2e.

Source Data Extended Data Figure 3.

Numerical data for Extended Data Figure 3f.

Source Data Extended Data Figure 4.

Numerical data for Extended Data Figure 4c.

Source Data Extended Data Figure 5.

Numerical data for Extended Data Figure 5b.
